# Vemurafenib inhibits necroptosis in normal and pathological conditions as a RIPK1 antagonist

**DOI:** 10.1038/s41419-023-06065-8

**Published:** 2023-08-24

**Authors:** Mayu Sun, Xueqi Ma, Wei Mu, Haonan Li, Xiaoming Zhao, Tengfei Zhu, Jingquan Li, Yongliang Yang, Haibing Zhang, Qian Ba, Hui Wang

**Affiliations:** 1 State Key Laboratory of Systems Medicine for Cancer, Center for Single-Cell Omics, School of Public Health, Shanghai Jiao Tong University School of Medicine, Shanghai, China; 2 CAS Key Laboratory of Nutrition, Metabolism and Food Safety, Shanghai Institute of Nutrition and Health, Chinese Academy of Sciences, Shanghai, China; 3 School of Bioengineering, Dalian University of Technology, Dalian, China

**Keywords:** Necroptosis, High-throughput screening

## Abstract

Necroptosis, a programmed cell death with necrotic-like morphology, has been recognized as an important driver in various inflammatory diseases. Inhibition of necroptosis has shown potential promise in the therapy of multiple human diseases. However, very few necroptosis inhibitors are available for clinical use as yet. Here, we identified an FDA-approved anti-cancer drug, Vemurafenib, as a potent inhibitor of necroptosis. Through direct binding, Vemurafenib blocked the kinase activity of receptor-interacting protein kinases 1 (RIPK1), impeded the downstream signaling and necrosome complex assembly, and inhibited necroptosis. Compared with Necrostain-1, Vemurafenib stabilized RIPK1 in an inactive DLG-out conformation by occupying a distinct allosteric hydrophobic pocket. Furthermore, pretreatment with Vemurafenib provided strong protection against necroptosis-associated diseases in vivo. Altogether, our results demonstrate that Vemurafenib is an effective RIPK1 antagonist and provide rationale and preclinical evidence for the potential application of approved drug in necroptosis-related diseases.

## Introduction

Necroptosis is a programmed cell death that is characterized by cellular swelling, plasma membrane rupture, and subsequent release of intracellular components [1]. Unlike unordered necrosis, necroptosis is a more physiological and regulated type and is dependent on the activation of receptor-interacting protein kinases (RIPKs). Increasing evidence suggests that necroptosis is implicated in mediating multiple human diseases, including systemic inflammation, neurodegeneration, autoimmune diseases, and cancer [2–5]. Therefore, interventions targeting the necroptosis signaling pathway may be a promising therapeutic strategy for necroptosis-associated diseases.

Necroptosis can be triggered by various stimuli, such as tumor necrosis factor α (TNFα), interferon γ, lipopolysaccharide (LPS), and viral nucleic acids [6, 7]. Among them, TNFα-initiated necroptosis is the most extensively studied model. In response to TNFα stimulation, RIPK1 is recruited to the cytoplasmic domain of TNFR1 and initiates the formation of protein complex I [8]. Under the condition of caspase-8 inactivation, RIPK1 can interact with RIPK3 through their RIP homotypic interaction motifs (RHIM) to form a necrosome, and promote the autophosphorylation and activation of RIPK3 [9–11]. RIPK3 then recruits and phosphorylates the downstream effector, mixed lineage kinase domain-like (MLKL), which executes necroptosis by translocating to the plasma membrane and triggering its rupture [12, 13]. During this process, activation of RIPK1, RIPK3, and MLKL is essential for TNFα-induced necroptosis, thus, inhibition of these signaling proteins can protect cells from necroptosis.

The activation loop for protein kinases is characterized by a conserved DFG motif (DLG-motif for RIPK1), which can regulate protein kinase to adopt two different conformations, active DFG-in or inactive DFG-out, and function as the molecular switch [14]. To date, most RIPK1/3 kinase inhibitors are designed to fall into two categories according to their binding mode: Type II and Type III [15]. Type II kinase inhibitors bind to the DFG-out inactive conformation and occupy both the allosteric hydrophobic pocket and the hinge region, whereas type III kinase inhibitors target the DFG-out conformation by only binding to the allosteric hydrophobic pocket [15]. The first small molecular inhibitor of necroptosis, Necrostatin-1 (Nec-1), belongs to type III kinase inhibitor [16, 17]. As the canonical inhibitor of RIPK1 kinase activity, Nec-1 and its optimized analog Nec-1s have been widely used in mouse disease models to define the role of RIPK1 and necroptosis. However, due to its metabolic instability and off-target effects [18], Nec-1 is limited for further application in drug development. Currently, although several necroptosis inhibitors were reported, none have been approved for clinical use. For example, Ponatinib and Pazopanib inhibited necroptosis in cell models, the unknown effects in vivo and serious safety concerns limited their clinical application [19, 20]. New RIPK1 inhibitors such as GSK2982772 and GSK963 exhibited high potency in human cells but low efficacy in mouse and rat cells, limiting the evaluation in animal disease models [21, 22]. Therefore, it is urgent to discover novel necroptosis inhibitors that have the potential to achieve clinical benefits as soon as possible.

Besides structure-based inhibitor design, drug repositioning is a feasible strategy to address unmet clinical needs and offers new perspectives for the development of novel drug-like inhibitors of necroptosis. In this study, we performed a cellular screening with an FDA-approved drug library and identified B-Raf^V600E^ inhibitor, Vemurafenib, as a novel necroptosis inhibitor. Through binding the kinase domain of RIPK1, Vemurafenib inhibited the kinase activity of RIPK1, blocked the activation of downstream signaling, and suppressed necroptosis in cells and mice. Notably, Vemurafenib bound to RIPK1 in a distinct form with Nec-1. By inhibiting necroptosis, Vemurafenib effectively protected from necroptosis-associated diseases in vivo. Our findings implicate a new function of Vemurafenib and raise the possibility that vemurafenib may serve as an option for treatment of RIPK1/necroptosis-mediated diseases.

## Materials and methods

### Mice and cell lines

C57BL/6 male mice, 8 weeks old, were purchased from Shanghai Slac Laboratory Animal and maintained in a pathogen-free vivarium under standard conditions. The animal study was reviewed and approved by the Institutional Animal Care and Use Committee of Shanghai Jiao Tong University School of Medicine. All mice experiments were carried out randomized and performed blinded. L929, MDF, and HT29 cells were cultured in DMEM medium supplemented with 10% fetal bovine serum, 100 U/mL penicillin, and 100 µg/mL streptomycin. The cell lines used in our study were tested to be free of mycoplasma contamination. Cell viability was assessed by CellTiter-Glo® Luminescent Cell Viability Assay kit following the manufacturer’s instructions (Promega).

### Antibodies and reagents

The antibodies used for immunobloting were anti-human-Phospho-RIPK1 antibody (65746; Cell Signaling); anti-mouse-Phospho-RIPK1 antibody (31122; Cell Signaling); anti-human/mouse-RIPK1 antibody (3493; Cell Signaling); anti-human-Phospho-RIPK3 antibody (93654; Cell Signaling); anti-mouse-Phospho-RIPK3 antibody (57220; Cell Signaling); anti-human-RIPK3 antibody (13526; Cell Signaling); anti-mouse-RIPK3 antibody (95702; Cell Signaling); anti-human-Phospho-MLKL antibody (91689; Cell Signaling); anti-mouse-Phospho-MLKL antibody (37333; Cell Signaling); anti-human-MLKL antibody (ab184718; Abcam); anti-mouse-MLKL antibody (89521-550; Abgent); anti-IκBα antibody (9242; Cell Signaling); anti-Phospho-IκBα antibody (2859; Cell Signaling); anti-Phospho-NF-κB P65 antibody (3033; Cell Signaling); anti-NF-κB P65 antibody (4764; Cell Signaling); anti-B-Raf antibody (ab200535; Abcam); anti-Flag M2 antibody (F1804; Sigma Aldrich); anti-β-Actin antibody (3700; Cell Signaling); anti-GAPDH antibody (2118; Cell Signaling); anti-rabbit IgG antibody (5127; Cell Signaling). The anti-human/mouse RIPK1 antibody used for immunoprecipitation was purchased from BD Biosciences (610459). The fluorochrome-labeled anti-mouse antibodies used for flow cytometry measurements were BV510-CD45 (563891; BD Biosciences); PE-Cy7-CD11b (552850; BD Biosciences); BV421-F4/80 (123132; Biolegend). The reagents used were Necrostatin-1 (T1847; Target Mol); Vemurafenib (T2382; Target Mol); z-VAD-fmk (T6013; Target Mol); SMAC mimetic BV6 (S7597; Selleck); Human TNFα recombinant protein (8902; Cell Signaling); mouse TNFα recombinant protein (24095; Cell Signaling).

### In vitro high-throughput drug screens

A commercial FDA-approved drug library (1068 compounds) was purchased from Target Mol (L4200). The screening was performed by assessing the inhibitory effect of chemicals on TNF-induced necroptotic cell death. L929 cells (1 × 10^4^ per well) were plated in 96-well plate and treated with TNFα (20 ng/mL) and 10 µM of chemicals. Each plate contained a negative control (dimethyl sulfoxide) and a positive control (Nec-1, 10 µM). 24 hours after incubation, cell viability was examined by using CellTiter-Glo® Luminescent Cell Viability Assay kit. The experiments were repeated three times independently and data were recorded as the mean.

### Immunoblotting and immunoprecipitation

Cultured cells were lysed with RIPA buffer with freshly added protease inhibitor cocktail. After incubation on ice, cell lysates were centrifuged and the protein concentration was determined by Pierce BCA Assay Kit (Thermo Fisher Scientific). The supernatants were collected and heated to 95 °C in Protein Loading Buffer (TransGen) for 8 mins. The proteins were resolved by polyacrylamide gel electrophoresis and transferred to PVDF membrane (Millipore). The membranes were then blocked with 5% skim milk in Tris-buffered saline plus 0.1% Tween 20 (TBST) for 1 hour at room temperature and blotted with primary antibodies overnight at 4 °C. After washing the membrane with TBST three times, the membranes were incubated with horseradish peroxidase–conjugated secondary antibodies for 2 hours. The bands were detected using Tanon-5200 Chemiluminescent Imaging System (Tanon Science & Technology).

For detecting RIPK3 and MLKL oligomerization, cells were lysed with non-reducing sample buffer and analyzed by immunoblotting. For immunoprecipitation, cell lysates were centrifuged and the supernatants were incubated with indicated antibodies overnight at 4 °C followed by 5 hours of incubation with 40 μl of Protein A/G agarose beads (Millipore). Beads were washed five times and proteins were eluted by boiling with 2X SDS sample buffer for 8 mins.

### In vitro kinase activity assay

Flag-tagged human RIPK1 was transfected in HEK293T cells for 72 hours. Cells were lysed with RIPA buffer with freshly added protease inhibitor cocktail (Cell Signaling). The supernatants were incubated with anti-Flag beads overnight at 4 °C. Beads were washed five times and RIPK1 proteins were eluted with Flag peptide. For the in vitro kinase activity assay, RIPK1 proteins were preincubated with DMSO or Vemurafenib for 30 mins. The kinase activity of RIPK1 was measured by ADP-Glo Kinase Assay kit following the manufacturer’s instructions (Promega).

### Molecular docking

The crystallographic structure of RIPK1 was obtained from the PDB database (https://www.rcsb.org/) (PDB code: 5HX6 and 4ITH). All the possible ligand-binding sites of RIPK1 were calculated and determined by FTSite tools [23] (https://ftsite.bu.edu/). The complex structures of Vemurafenib-RIPK1 and Nec-1-RIPK1 were computed via our in-house developed tool FIPSDock [24] and subsequently the solvated complexes were used as the initial structures for 100 ns MD simulations employing the amber 99 sb force field. The MD simulations were conducted by Gromacs 2021.1 tools. Furthermore, root-mean-square deviation (RMSD) and root-mean-square fluctuation (RMSF) calculation were conducted. The MM-PBSA calculation was performed using the gmx_mmpbsa of GROMACS. All molecular graphics and the putative contacts between RIPK1 and compounds were monitored over the course of the entire simulations by the PyMOL educational version (Invoice, #36011).

### Drug affinity response target stability assay

Cells were lysed with RIPA buffer with freshly added protease inhibitor cocktail. Cell lysates (3 mg/mL) were incubated with DMSO or Vemurafenib for 30 mins at room temperature. Following the incubation, each sample was digested with 0.1-1 μg/mL pronase (MedChemExpress) for 30 mins at room temperature. Digestion of lysates was stopped by the addition of SDS loading buffer and then boiling at 95 °C for 8 mins. Samples were loaded into SDS-PAGE gels for immunoblotting.

### Cellular thermal shift assay

Cells were treated with DMSO or Vemurafenib at 30 μM for 4 hours, and then harvested and subsequently resuspended in PBS containing protease inhibitor cocktail. The cell suspensions were aliquoted into 10 PCR tubes and heated to different temperatures for 3 mins followed by incubation at room temperature for 3 mins. Then, cells were lysed by 3 repeated freeze-thaw cycles using liquid nitrogen and centrifuged at 20,000 *g* for 20 mins at 4 °C. The supernatants were collected and analyzed by immunoblotting.

### Flow cytometric analyses of CD11b^+^F4/80^+^ peritoneal macrophages

8-week-old C57BL/6 mice were pretreated with Nec-1 (1.66 mg/kg body weight) or Vemurafenib (5 mg/kg body weight) via intraperitoneal injection for 15 mins. Mice subsequently were injected intraperitoneally with z-VAD (20 mg/kg) for 1 hour before intraperitoneal injection with LPS (10 mg/kg). Mice were sacrificed 24 hours after z-VAD injection, resident peritoneal cells were isolated by flushing the peritoneal cavity with a single injection of 8 ml sterile PBS, and blood was collected. Peritoneal cells were immunostained and analyzed by flow cytometry. The serum concentration of IFNγ, TNFα, IL-1β, and MCP-1 was measured by enzyme-linked immunosorbent assay.

### TNFα-induced SIRS model

8-week-old C57BL/6 mice were pretreated with vehicle or Vemurafenib (5 mg/kg body weight) via intraperitoneal injection for 15 mins before intravenous administration of mTNFα (9 μg per mouse). Mice mortality and body temperature were continuously recorded after TNFα administration.

### Cerulein-induced pancreatitis

8-week-old C57BL/6 mice were pretreated with Nec-1 (1.66 mg/kg body weight) or Vemurafenib (5 mg/kg body weight) via intraperitoneal injection for 15 mins. Mice subsequently were injected intraperitoneally with saline or cerulein (MedChemExpress) at a concentration of 50 μg/kg body weight every 2 hours for 10 hours (total 6 injections). Mice were sacrificed after 2 hours of recovery. The pancreas was collected for H&E staining, and blood was collected for analysis of serum amylase and cytokines.

### Statistical analysis

All experiments were repeated at least three times. All values were presented as the means ± SEM. The data were analyzed by using GraphPad Prism V.7 (GraphPad Software). Statistical significance was determined through unpaired Student’s *t*-test or log-rank (Mantel-Cox) test and was considered significant when *p* < 0.05.

## Results

### Identification of Vemurafenib as a necroptosis inhibitor

**Fig. 1:**
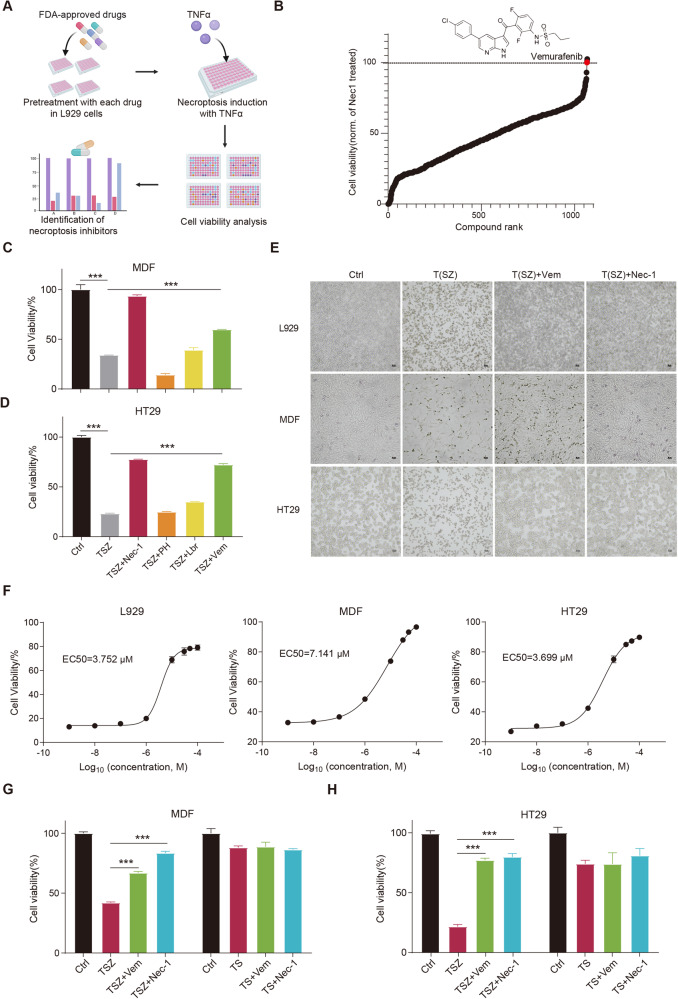
Identification of Vemurafenib as a new necroptosis inhibitor. **A** Scheme of the in vitro high-throughput drug screen workflow. L929 cells were treated with each compound (10 µM) and then stimulated with TNFα (20 ng/mL) for 24 hours. Cell viability was determined by CellTiter-Glo® assay. **B** Identification of necroptosis inhibitors by cellular screen with drug library. Normalized necroptosis inhibition efficiency for drugs tested depicted as a percentage of control (Nec-1 = 100). Data are represented as the mean of triplicates. The chemical structure of Vemurafenib is shown above. MDF (**C**) and HT29 (**D**) cells were pretreated with Promethazine hydrochloride (10 μM; PH), Lbrutinib (10 μM; Lbr) or Vemurafenib (10 μM; Vem) followed by treatment with TNFα (20 ng/mL), Smac mimetic (1 μM), and z-VAD (20 μM; TSZ) for 6 and 12 hours, respectively. Cell viability was determined by CellTiter-Glo® assay. **E** Representative images of L929, MDF, and HT29 cells treated with DMSO, Vemurafenib, or Nec-1 followed by necroptosis induction. Necroptosis in L929 cells was induced by TNFα for 24 hours. Necroptosis in MDF and HT29 cells was induced by TNFα, Smac mimetic, and z-VAD for 6 and 12 hours, respectively. **F** Dose-response curves showing the inhibitory effect of Vemurafenib on necroptosis in L929, MDF, and HT29 cells. MDF (**G**) and HT29 (**H**) cells were pretreated with DMSO, Vemurafenib (30 μM), or Nec-1 (10 μM) followed by indicated treatment for 6 and 12 hours, respectively. Cell viability was determined by CellTiter-Glo® assay. TS: TNFα (20 ng/mL) plus Smac mimetic (1 μM). *P* value was calculated by unpaired Student’s *t*-test. (*p < 0.05, **p < 0.01, ***p < 0.001).

To identify novel necroptosis inhibitors for potential clinical application, we screened the FDA-approved drug library using a well-established necroptosis model in mouse fibrosarcoma L929 cells (Fig. 1A), which are sensitive to TNFα-induced necroptosis even in the absence of caspase inhibitors [6, 25]. Nec-1, but not apoptosis inhibitors z-VAD, protected TNFα-induced cell death (Fig. S1A), confirming necroptosis rather than apoptosis in L929 cells. For drug screening, Nec-1 was used as a reference to identify positive compounds. Among the 1068 drugs, three drugs (Lbrutinib, Promethazine hydrochloride, and Vemurafenib) showed comparable or higher necroptosis-rescuing effects than Nec-1 (Fig. 1B), which were further validated in murine dermal fibroblast MDF and human colon cancer cells HT29. Unlike L929 cells, both MDF and HT29 cells require additional interventions such as smac mimetic (S) and z-VAD (Z) for the induction of necroptosis in response to TNFα (T) stimulation. Notably, our results indicated that only Vemurafenib significantly inhibited the TSZ-induced necroptosis as Nec-1 in both cell models (Fig. 1C, D). Besides, in all three cell models, Vemurafenib prevented TNFα- or TSZ-induced necrotic morphology, such as cell swelling and plasma membrane rupture (Fig. 1E). The inhibitory activity of Vemurafenib was also found in TNFα, cycloheximide, and z-VAD (TCZ)-induced necroptosis (Fig. S1B), further supporting the suppression of Vemurafenib on necroptosis. To quantitatively assess the inhibitory potency of Vemurafenib, we performed a dose-response assay and found that Vemurafenib dose-dependently inhibited TNFα- or TSZ-induced necroptosis in L929, MDF, and HT29 cells, with an EC_50_ of ~3.752, 7.141, and 3.699 μM, respectively (Fig. 1F). Notably, Vemurafenib did not inhibit apoptosis induced by TNFα plus smac mimetic (TS) in MDF and HT29 cells, indicating the specificity of Vemurafenib on necroptotic cell death (Fig. 1G, H). Taken together, these results demonstrate that Vemurafenib is a potent and selective inhibitor of necroptosis induced by various stimuli in both human and mouse cells.

### Vemurafenib blocks RIPK1 activation and disrupts necrosome formation

**Fig. 2:**
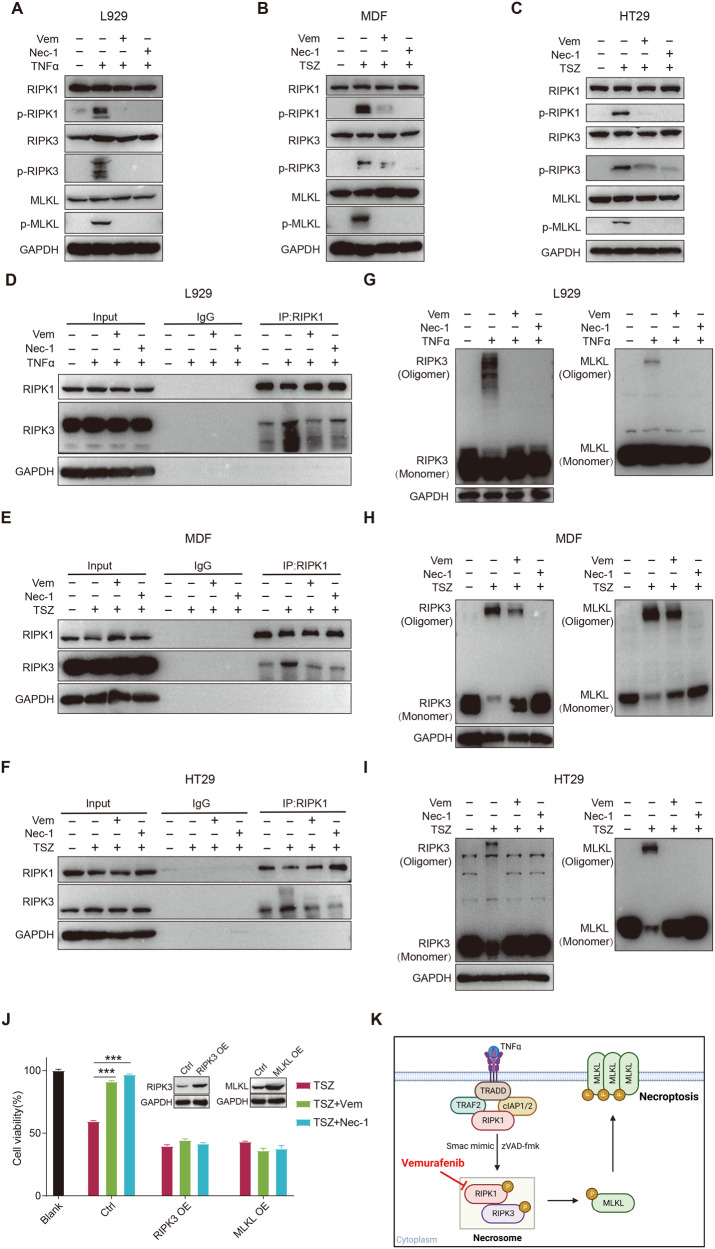
Vemurafenib inhibits necroptotic pathway and blocks necrosome formation. L929 (**A**), MDF (**B**), and HT29 (**C**) cells were pretreated with DMSO, Vemurafenib (30 μM; Vem) or Nec-1 (10 μM) followed by necroptosis induction for 12, 3, and 8 hours, respectively. The protein levels were determined by western blot. Immunoprecipitates of RIPK1 from L929 (**D**), MDF (**E**), and HT29 (**F**) cells after indicated treatments were subjected to western blot to detect the associations with RIPK1 and RIPK3. Cell lysates from L929 (**G**), MDF (**H**), and HT29 (**I**) cells after indicated treatments were subjected to nonreducing SDS/PAGE and detected the RIPK3 and MLKL oligomer. **J** MDF cells were transfected with RIPK3 or MLKL for 48 hours. Then cells were pretreated with Vemurafenib or Nec-1 followed by necroptosis induction. Cell viability was determined by CellTiter-Glo® assay. **K** Schematic depiction of the inhibitory effect of Vemurafenib on TNFα-induced necroptosis. *P* value was calculated by unpaired Student’s *t*-test. (*p < 0.05, **p < 0.01, ***p < 0.001).

To investigate the molecular mechanism of Vemurafenib against necroptotic cell death, we first examined whether NF-κB, the canonical downstream of TNFα, mediated the protection of Vemurafenib. Similar to Nec-1, Vemurafenib did not affect the activation of NF-κB and IκB-α after TNFα or TSZ stimulation (Fig. S2A), ruling out the involvement of NF-κB signaling pathway. We then detected the effects of Vemurafenib on the major steps of necroptotic signaling. As an upstream mediator of the necroptosis signaling cascade, RIPK1 kinase is required to form necrosome and initiate necroptosis. After necroptotic stimulation, RIPK1, as well as the downstream proteins RIPK3 and MLKL, were significantly phosphorylated, while the addition of Vemurafenib or Nec-1 almost completely abolished the activation of these proteins among all cell models (Fig. 2A–C). Next, we investigated the formation of necrosome. After treatment with Vemurafenib, the interaction between RIPK1 and RIPK3 was significantly blocked, suggesting the disruption of necrosome formation by Vemurafenib (Fig. 2D–F). RIPK3 in the necrosome can recruit free RIPK3 to form RIPK3 oligomerization, which further promotes MLKL oligomerize and translocate to the plasma membrane for rupture [26]. We found that Vemurafenib largely diminished the oligomerization of both RIPK3 and MLKL (Fig. 2G–I), suggesting dysfunctional downstream events. Overexpression of these downstream proteins can be sufficient to induce necroptosis bypassing the upstream signaling [27, 28]. Notably, the inhibitory effect of Vemurafenib was lost for necroptosis after overexpression of RIPK3 or MLKL (Fig. 2J). Also, Vemurafenib did not influence the autophosphorylation of RIPK3 in overexpression system (Fig. S2B), suggesting that Vemurafenib targeted upstream necroptotic pathway (RIPK1 activation) (Fig. 2K).

Vemurafenib was approved for treatment of metastatic and advanced BRAF-mutated melanoma as the first selective BRAF kinase inhibitor [29]. By binding to the ATP-binding site of mutant BRAF(V600E) kinase, Vemurafenib inhibits the active DFG conformation and inactivates the kinase activity [30]. To identify the role of BRAF in the necroptosis-inhibiting effects of Vemurafenib, we silenced BRAF using siRNA in MDF cells (Fig. S3A). Interestingly, BRAF knockdown did not affect the necroptotic cell death and the activation of RIPK1 and MLKL after TSZ stimulation (Fig. S3A, B). Also, the knockdown of BRAF had no obvious effects on the protection of both Nec-1 and Vemurafenib against necroptosis (Fig. S3A, B), thus excluding the involvement of BRAF in necroptosis signaling.

Overall, these results demonstrate that Vemurafenib blocks necroptosis by inhibiting the phosphorylation of RIPK1 and disturbing the formation of necrosome.

### Vemurafenib is a novel antagonist of RIPK1 kinase

**Fig. 3:**
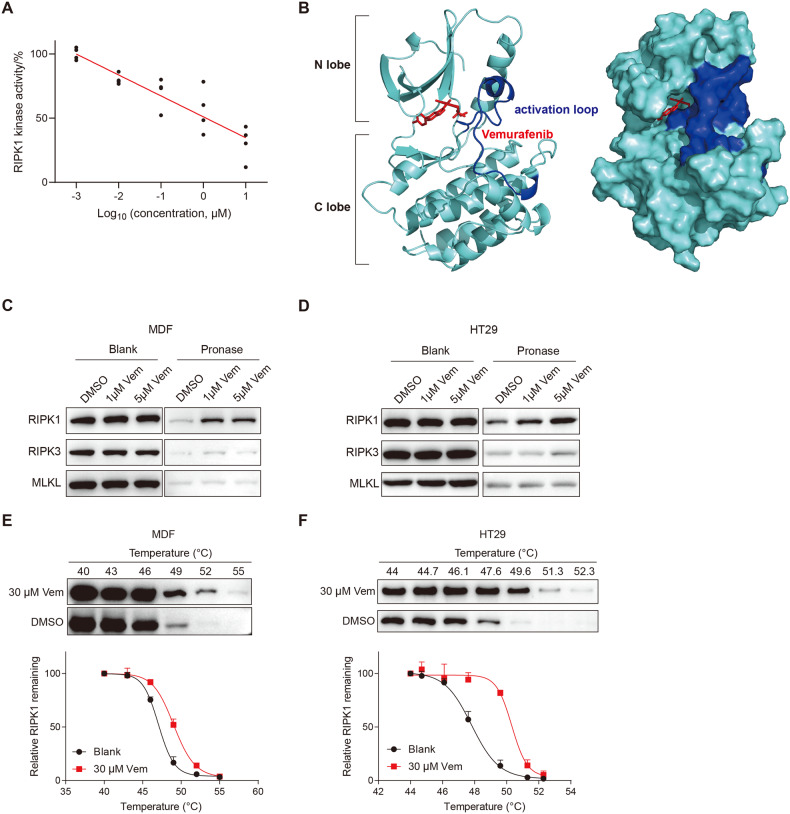
Vemurafenib directly binds to inhibit the activity of RIPK1. **A** In vitro kinase activity assay using recombinant RIPK1 proteins. Human recombinant RIPK1 proteins were purified from transfected HEK293T cells and the kinase activity was measured after preincubation with Vemurafenib for 30 mins. **B** Cartoon (left) and surface mode (right) of computationally optimized overall structure of Vemurafenib-bound RIPK1. The human RIPK1 structure was obtained from Protein Data Bank (PDB: 5XH6). The N- and C-lobes of RIPK1 kinase domain are colored cyan. The activation loop (residues Asp156–Glu196) is colored blue. Vemurafenib is colored red. All structural figures were prepared with PyMOL. Cell lysates of MDF (**C**) and HT29 (**D**) cells were incubated with DMSO or Vemurafenib (Vem) for 30 mins followed by treatment with pronase for 30 mins. The proteolysis rates of RIPK1, RIPK3, and MLKL were determined by western blot. MDF (**E**) and HT29 (**F**) cells were preincubated with DMSO or Vemurafenib and then subjected to CETSA assay to detect the thermal stability of RIPK1. Upper panel: the western blot images for RIPK1; Lower panel: quantification of the band intensities.

Based on the aforementioned findings, we proposed that Vemurafenib may directly target and inhibit RIPK1. To test this hypothesis, firstly we performed in vitro kinase activity assay to test whether Vemurafenib inhibits the activity of RIPK1 directly. Incubation of recombinant human RIPK1 with Vemurafenib led to a strong decrease of the kinase activity of RIPK1 in a dose-dependent manner (Fig. 3A), suggesting a direct interaction between Vemurafenib with RIPK1. Next, we conducted a molecular docking study to model the interaction based on the cocrystal structure of RIPK1 (PDB code 5HX6). RIPK1 exhibits a canonical kinase fold, with an N-lobe, a C-lobe, and an intervening regulatory activation loop (residues 156-196, also known as the T-loop) [17]. The simulation results showed that Vemurafenib bound preferably to the hydrophobic pocket between the N-lobe and C-lobe at the back of the ATP binding site, in close proximity to the activation loop (Fig. 3B). In this binding mode, the propyl tail group of Vemurafenib was inserted into an interior hydrophobic cavity formed by activation loop, which could lock RIPK1 kinase in an inactive DLG-out conformation without interaction with the hinge region, suggesting a possible allosteric inhibitory mechanism. Furthermore, we verified the direct interaction between Vemurafenib and RIPK1 by the drug affinity response target stability (DARTS) and cellular thermal shift assay (CETSA). In DARTS, Vemurafenib effectively protected RIPK1 from degradation by pronase (Fig. 3C, D). In contrast, the proteolysis of RIPK3 and MLKL was not affected in the presence of Vemurafenib (Fig. 3C, D), thereby again supporting the notion that Vemurafenib selectively binds to RIPK1, but not other necroptotic proteins. In CETSA, incubation with Vemurafenib resulted in a substantial and enhanced shift of the thermal stability of RIPK1 (Fig. 3E, F), suggesting that Vemurafenib is able to bind endogenous RIPK1 in situ. Taken together, these data suggest that Vemurafenib direct binds and antagonizes kinase activity of RIPK1.

### A unique binding mode of Vemurafenib to RIPK1 kinase

The N-terminal kinase domain of RIPK1 is known to present several essential residues and display kinase-dependent and -independent functions in regulating TNF-mediated necroptosis [16, 31], we thus validated whether Vemurafenib binds to the kinase domain. We constructed the Flag-tagged full-length or kinase domain (residues 1-330) of human RIPK1 and found that Vemurafenib effectively inhibited the auto-phosphorylation of both full-length and fragmented RIPK1 (Fig. S4A). Moreover, the thermal stability of kinase domain RIPK1 fragment was significantly increased after Vemurafenib treatment (Fig. S4B), confirming the direct binding of Vemurafenib to kinase domain.

**Fig. 4:**
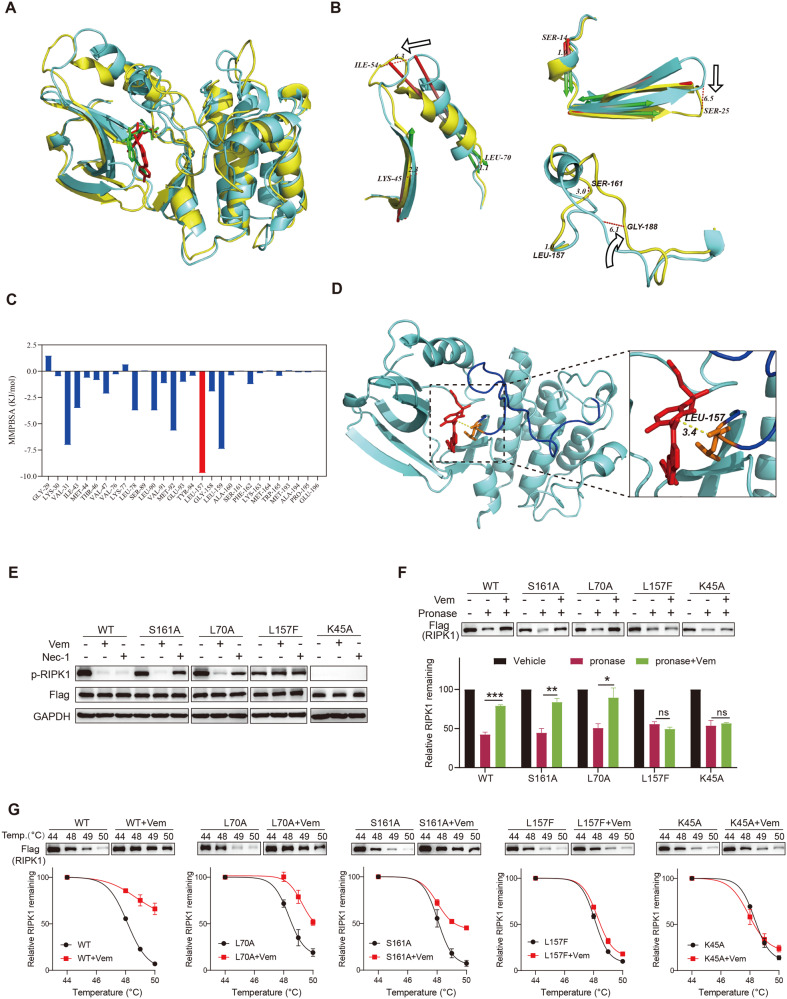
Identification of Vemurafenib-binding sites in RIPK1 kinase. **A** The structural alignment of the initial (cyan cartoon) and final (yellow cartoon) conformations of Vemurafenib-bound RIPK1 during 100 ns MD simulations. The initial and last frames of Vemurafenib binding position were shown by red and green sticks. **B** Alignment of the initial (cyan cartoon) and final (yellow cartoon) conformations of the helix (residues 37–66), beta-strand (residues 10–36), and activation loop (residues 156–196) of Vemurafenib-bound RIPK1. Movement of helix, beta-strand, and activation loop is indicated by arrow. **C** Analysis of binding free energy contribution (MM/PBSA model) of key residues in the binding pocket. **D** Close-up view of the interaction between Vemurafenib and residue Leu157 of RIPK1. The orange stick structure represents Leu157 and the red stick represents Vemurafenib. The cyan cartoon represents the RIPK1 in the last frame of MD simulations. The yellow line indicates the possible electrostatic contacts between Leu157 of RIPK1 with Vemurafenib. **E**–**G** HEK293T cells were transfected with Flag-tagged WT, S161A mutant, L70A mutant, L157F mutant, and K45A mutant of RIPK1, respectively. After indicated treatment, the protein levels and phosphorylation of RIPK1 (**E**) were determined by western blot; the proteolysis rate of RIPK1 (**F**) was determined by DARTS Assay; the thermal stability of RIPK1 (**G**) was determined by CETSA assay. *P* value was calculated by unpaired Student’s *t*-test. (*p < 0.05, **p < 0.01, ***p < 0.001).

We then conducted the 100 ns molecular dynamics (MD) simulations to investigate the possible binding modes and action mechanisms. The root mean square deviation (RMSD) and root mean square fluctuation (RMSF) of Vemurafenib and RIPK1 relative to the initial structure were calculated to evaluate the binding stability and flexibility. As shown in Fig. S4C, D, the conformation of RIPK1 with Vemurafenib reached equilibrium after 10 ns, and the RMSF values of most residues of RIPK1 were below 2 Å, suggesting that Vemurafenib forms stable complexes with RIPK1 throughout the whole MD simulations process. During the 100 ns MD simulations, we found that Vemurafenib occupied a putative allosteric binding pocket (displayed in yellow mesh) adjacent to but distinct from the conventional kinase domain (displayed in pink mesh) which Nec-1 bound (Fig. S4E). Compared to Nec-1 which bound to the deep inside part of the allosteric pocket, Vemurafenib bound extendedly to the outside part of the pocket surrounded by the five-stranded β sheet of N-lobe and activation loop. These residues located in the five-stranded β sheet and activation loop could have higher probabilities to form interactions with Vemurafenib. Alignment of the two complex structures before and after 100 ns MD simulations revealed that Vemurafenib can make a significant impact on the conformation change of RIPK1, especially the β strand (residues 10–36) and helix (residues 37–66) of N-lobe and activation loop (Fig. 4A, B). These conformation changes were also found in 100 ns MD simulations of Nec-1-bound RIPK1 (Fig. S4F, G).

To investigate the binding affinities between Vemurafenib and RIPK1 and identify the hotspot residues, we calculated the contribution of each residue to the binding free energy by molecular mechanics poisson-boltzmann surface area (MM-PBSA) method. The energy contribution analysis showed that the average MM-PBSA binding free energy of Vemurafenib with RIPK1 is −134.142 kJ/mol (Supplementary Table 1). Notably, Leu157 made the most significant contribution in the allosteric pocket (−9.7 kJ/mol) (Fig. 4C). Further analysis found that there were existing possible electrostatic contacts between Leu157 and Vemurafenib as displayed in Fig. 4D, indicating that Leu157 is a key residue for binding with Vemurafenib.

To further verify the Vemurafenib-binding mode proposed above with native RIPK1 in live cells, we performed site-directed mutagenesis. We selected the key residue Leu157 and the other two residues Leu70 and Ser161, which are crucial for binding to Nec-1 but not Vemurafenib in MM-PBSA model (Supplementary Table 1). Consistent with the structural interactions, L70A, L157F, and S161A mutations dramatically attenuated the inhibition in kinase activity of RIK1 by Nec-1, as indicated by the levels of autophosphorylation of RIPK1 (Fig. 4E). In contrast, Vemurafenib still effectively inhibited the autophosphorylation of L70A and S161A mutations (Fig. 4E). However, the inhibitory efficiency of Vemurafenib was lost against L157F mutation (Fig. 4E). Thus, our results confirm the different binding modes between Vemurafenib and Nec-1, and indicate that Leu70 and Ser161 are important for Nec-1, but not Vemurafenib, to inhibit RIPK1 kinase activity; whereas Leu157 is essential for both inhibitors. We further verified these results by DARTS and CETSA. As shown in Fig. 4F,G, Vemurafenib protected L70A and S161A mutations from protease degradation and increased the thermal stability of L70A and S161A mutants as with WT-RIPK1 (Fig. 4F, G). However, both protease degradation and thermal stability of RIPK1 were no longer affected by Vemurafenib when Leu157 was mutated (Fig. 4F, G). In addition, we found that K45A, a kinase-dead mutation (Fig. 4E) significantly blocked the interaction of Vemurafenib and RIPK1, indicating that Lys45 is also important for Vemurafenib to bind to RIPK1 (Fig. 4F–G).

Taken together, these results support our predicted binding mode of Vemurafenib to stabilize RIPK1 kinase in an inactive DLG-out conformation by occupying a distinct binding pocket, thus being indicative of a type III kinase inhibitor.

### Vemurafenib suppresses LPS/z-VAD-induced macrophage necroptosis in vivo

**Fig. 5:**
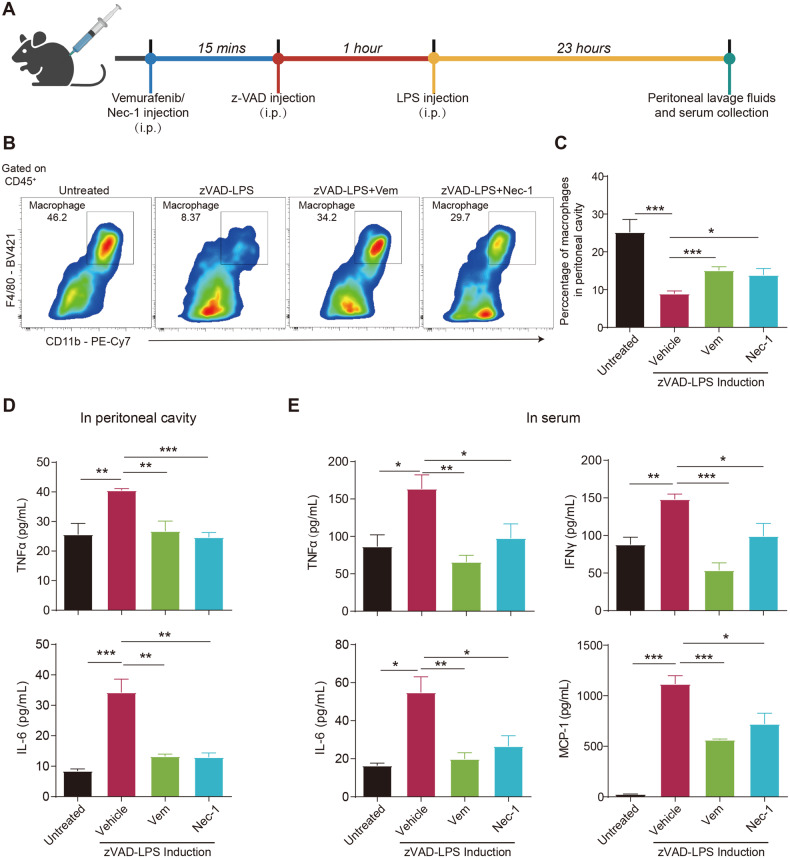
Vemurafenib alleviates LPS/z-VAD-induced macrophage necroptosis in mice. **A** Scheme of the murine model of macrophage necroptosis. **B**, **C** Representative flow cytometry plots and quantification showing the proportion of macrophages (CD11b^+^F4/80^+^) in the peritoneal cavity. *n* = 10 per group. The levels of proinflammatory cytokines in the peritoneal cavity (**D**) and serum (**E**) were determined by ELISA. *n* = 5 per group. *P* value was calculated by unpaired Student’s *t*-test. (*p < 0.05, **p < 0.01, ***p < 0.001).

We next examined the inhibitory effect of Vemurafenib on necroptosis in vivo. Previous studies have revealed that lipopolysaccharide (LPS) can induce necroptosis of intraperitoneal macrophages in the presence of z-VAD, whereas genetic inactivation or pharmacological inhibition of RIPK1 effectively prevented LPS/z-VAD-induced macrophage death [32–35]. We thus investigated the effect of Vemurafenib in this mouse model through pretreatment with Vemurafenib or Nec-1 for 15 mins following challenges with LPS/z-VAD (Fig. 5A). As expected, compared with control mice, LPS/z-VAD significantly reduced the percentage of macrophages in the abdominal cavity, while pretreatment with Vemurafenib, like Nec-1, attenuated the macrophage loss, indicating that Vemurafenib conferred macrophages resistance to necroptosis induced by LPS/z-VAD (Fig. 5B, C). Proinflammatory cytokines production is a consequence of macrophage necroptosis, which in turn promotes RIPK1-driven necroptosis [34]. We found that the elevated levels of TNFα and IL-6 in peritoneal fluid and TNFα, IL-6, IFN-γ, and MCP-1 in serum were significantly blocked after the treatment of Nec-1 or Vemurafenib (Fig. 5D, E). Therefore, Vemurafenib could inhibit LPS/z-VAD-induced macrophage necroptosis in vivo and reduce the secretion of proinflammatory cytokines.

### Vemurafenib protects mice from necroptosis-associated pathogenesis

**Fig. 6:**
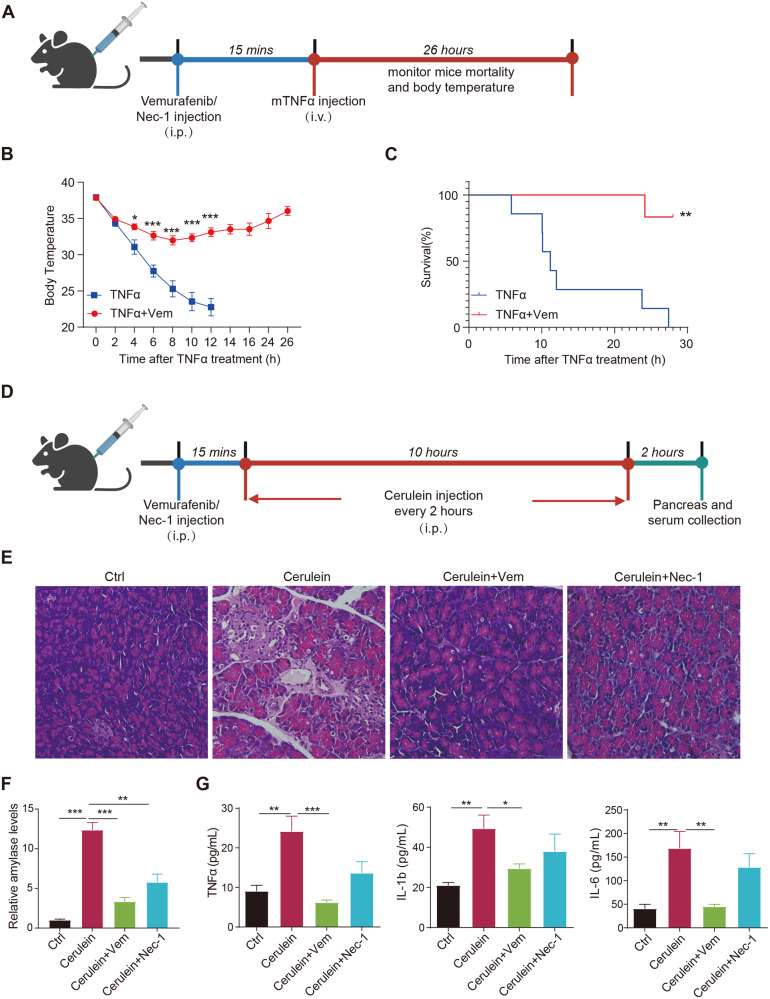
Vemurafenib protects against TNFα-induced SIRS and cerulein-induced pancreatitis. **A** Scheme of TNFα-induced SIRS model. The body temperature (**B**) and survival (**C**) of mice pretreated with Vemurafenib (Vem) were continuously recorded after TNFα injection. *n* = 6–7 per group. **D** Scheme of cerulein-induced pancreatitis. **E** H&E staining of the pancreas from mice treated with Vemurafenib or Nec-1 following caerulein injection. The serum levels of amylase (**F**) and proinflammatory cytokines (**G**) were determined after indicated treatments. *n* = 6 per group. *P* value in **B**, **F** and **G** was calculated by unpaired Student’s *t*-test. *P* value for survival curve was calculated by the log-rank (Mantel-Cox) test. (*p < 0.05, **p < 0.01, ***p < 0.001).

Necroptosis is involved in multiple human inflammatory diseases. To evaluate the therapeutic effect of Vemurafenib on necroptosis-associated diseases in vivo, we utilized the TNFα-induced systemic inflammatory response syndrome (SIRS) mice model (Fig. 6A), which is a RIPK kinase-driven disease and strongly prevented by inhibiting necroptotic pathway [2]. As expected, administration of mouse TNFα induced the characteristic drop in body temperature and resulted in the death of mice in 28 hours (Fig. 6B, C). In contrast, pretreatment with 5 mg/kg Vemurafenib for 15 mins provided potent protection against TNFα-induced acute hypothermia (Fig. 6B) and lethal septic shock, with only 17% of the mice succumbing (Fig. 6C).

Severe acute pancreatitis is an inflammatory disorder, which manifests with cell death and increased proinflammatory cytokines in serum and ultimately results in systemic inflammatory responses. Necroptosis has been reported to be the predominant type of acinar cell death in caerulein-induced acute pancreatitis [9, 36, 37]. The severity of caerulein-induced acute pancreatitis can be reduced by pharmacological inhibition of RIPK1 [36, 38–40]. We thus explored whether Vemurafenib could mitigate the tissue injury in caerulein-induced acute pancreatitis model (Fig. 6D). Compared with control group, caerulein caused severe pancreatic damage, whereas administration of Vemurafenib was able to functionally ameliorate the necrosis areas of acinar cells and the severity of histological damage (Fig. 6E). Consistently, serum amylase level, a diagnostic biomarker for acute pancreatitis, was markedly increased in caerulein-treated group, but recovered after addition with Vemurafenib, indicating the alleviation of acute pancreatitis by Vemurafenib (Fig. 6F). Furthermore, pretreatment with Vemurafenib significantly decreased the serum levels of caerulein-induced proinflammatory cytokines including TNFα, IL-β, and IL-6 (Fig. 6G). Vemurafenib produced the same effect as Nec-1, consistent with its mechanism of action against necroptosis.

These results indicate that Vemurafenib can confer effective protection against necroptosis-associated diseases in vivo.

## Discussion

Necroptosis has been found to be involved in the occurrence and development of a variety of diseases. However, to date, the pharmacological agents that specifically target necroptosis are still in clinical trials, and far from the application. This clinical status has inspired us and others to pursue the development of necroptosis inhibitors. Herein, we screened an FDA-approved drug library in order to discover potential inhibitors of necroptosis. From the results, we identified Vemurafenib as a novel necroptosis antagonist that selectively inhibits RIPK1 kinase activity and blocks necroptotic cell death both in vitro and in vivo.

Vemurafenib has previously been identified as a highly specific BRAF(V600E) kinase inhibitor and has been successfully used in the clinic to treat late-stage melanoma. However, the inhibition of BRAF by Vemurafenib may not contribute towards its protective effects against necroptosis observed here, as knockdown of BRAF did not affect activation of necroptosis induced by TSZ in MDF cells, indicating another mechanism independent of BRAF inhibition. The kinase domain of human RIPK1 shares strong sequence (28%) and structural similarities (47%) with that of BRAF [16, 17]. In particular, Ser161 autophosphorylation of RIPK1 in the activation segment corresponds to the Thr598 autophosphorylation of BRAF, which functions as an allosteric regulatory site that controls the kinase activity [16]. Most notably, consistent with the degree of sequence conservation, the crystal structure of Nec-1s bound to RIPK1 is also quite similar to that of Vemurafenib bound to BRAF [17], indicating that BRAF inhibitors may bind RIPK1 by targeting its kinase domain. Indeed, recent studies have shown that BRAF inhibitors Sorafenib and TAK-632, can bind to RIPK1 and induce an inactive conformation of RIPK1 [41, 42]. These studies suggest that RIPK1 may be another unknown target of Vemurafenib and required for Vemurafenib to inhibit necroptosis. Notably, it has been reported that BRAF inhibitors like Dabrafenib and Sorafenib can also bind to RIPK3 to block necroptosis [41, 43]. However, in our study, Vemurafenib did not show obvious interaction with the downstream protein RIPK3 and MLKL, displaying the high selectivity of Vemurafenib to RIPK1. This observation is consistent with a previous study demonstrating that Vemurafenib fails to block RIPK1-independent necroptosis in RIPK3-expressing cells [44], further supporting our proposed notion that Vemurafenib primarily inhibits necroptosis by binding to RIPK1, rather than RIPK3.

RIPK1 has emerged as an important upstream regulator that controls cell survival, inflammation, or cell death [45, 46]. As a scaffold protein, RIPK1 can promote cell survival by activating NF-κB signaling, while it also triggers cell death depending on its kinase activity. However, dysregulation of these RIPK1-mediated crucial cellular decisions can disrupt normal homeostasis and result in disease. Given the high levels of RIPK1 expression in many diseased tissues, RIPK1 has been recognized as a promising therapeutic target for the treatment of a wide range of human diseases. Currently, almost all the reported RIPK1 inhibitors target the conserved and unique hydrophobic pocket, located in the allosteric regulatory domain, and stabilize RIPK1 in a DLG-out inactive conformation. Similarly, our molecular docking study revealed that Vemurafenib, as a type III kinase inhibitor, bound to the allosteric hydrophobic pocket adjacent to the activation loop of RIPK1. By binding to the kinase domain, Vemurafenib suppressed RIPK1 autophosphorylation and subsequent formation of necrosome, and eventually blocked RIPK1-RIPK3-MLKL signal transduction and necroptotic cell death. Interestingly, the binding mode of Vemurafenib did not affect the scaffolding function of RIPK1, as Vemurafenib had no effect on NF-κB activation.

Nec-1 is the first small molecular inhibitor of necroptosis originally identified in a chemical library screen in 2005 [47] and was found to be a RIPK1-targeting inhibitor in further studies. Alignment of the Nec-1-bound RIPK1 with Vemurafenib-bound RIPK1 predicted structure revealed that Vemurafenib shares a portion of binding regions in the allosteric hydrophobic pocket of RIPK1 with Nec-1. The similarities and differences between the two binding modes explained their common binding sites (e.g., residues Leu157) and different binding sites (e.g., residues Leu70 and Ser161) in RIPK1. Notably, Vemurafenib and Nec-1 did not exhibit synergistic inhibitory effects in necroptosis models (data not shown). This may be attributed to the fact that both Vemurafenib and Nec-1 target the kinase domain of RIPK1 protein to inhibit necroptosis, resulting in overlapping or similar inhibitory effects. Although Nec-1 shows remarkable inhibitory effects on RIPK1-dependent necroptosis, its poor pharmacodynamic profiles limit further clinical applications. Unlike Nec-1, Vemurafenib is an orally available FDA-approved drug with high absorption and metabolic stability in vivo [48]. In the clinical setting, the recommended dose of Vemurafenib for treating metastatic melanoma with BRAF (V600E) mutation is 960 mg twice daily [49]. At this dose, the plasma levels of Vemurafenib at steady-state were reported to be 86 ± 32 μM [49]. This suggests that concentrations of 10–30 µM in cell culture and 5 mg/kg in mice, as used in our study, should be relatively safe and feasible. By targeting RIPK1-driven necroptotic pathway, Vemurafenib alleviated TNFα-induced systemic inflammation and caerulein-induced pancreatitis in murine disease models. It has been shown that necroptosis of solid tumors is often associated with tumor metastasis and is considered a poor prognostic feature for metastatic tumors [50–52]. Necroptosis blockade by Nec-1 substantially decreases tumor cell extravasation and metastasis in a melanoma metastatic model [53]. Given that Vemurafenib has significant clinical efficacy in metastatic melanoma, its potent anti-tumor effects may, besides the direct cytotoxicity on tumor cells, be attributed to the inhibition of necroptosis.

In summary, this study identified an FDA-approved drug, Vemurafenib, as a novel and effective antagonist of RIPK1 kinase, and determined the effective protection of vemurafenib against necroptosis and associated diseases. These findings not only provide preclinical evidence and rationale for necroptosis-targeting therapeutic strategy in inflammatory disorders, and also offer an approved drug candidate with broad application prospects.

## Supplementary information

Supplementary figure and table legends

Supplementary Figure 1

Supplementary Figure 2

Supplementary Figure 3

Supplementary Figure 4

Supplementary table 1

original western blots

## Data Availability

All data relevant to the study are included in the article or uploaded as supplementary information.
