# Supplementary material for: Vemurafenib inhibits necroptosis in normal and pathological conditions as a RIPK1 antagonist
Source: Cell Death Dis. 2023 Aug 24;14(8):555. doi: 10.1038/s41419-023-06065-8 (PMC10449909; doi:10.1038/s41419-023-06065-8)
Supplement: Supplementary file 1 — Supplementary figure and table legends [file 41419_2023_6065_MOESM1_ESM.docx]

**Supplementary Figure 1.** (**A**) After indicated treatment, the viability of L929 cells was determined by CellTiter-Glo® assay. (**B**) MDF and HT29 cells were pretreated with DMSO, Vemurafenib (Vem), or Nec-1 followed by treatment with TNFα (20 ng/mL), cycloheximide (5 µg/mL), and z-VAD (20 μM; TCZ) for 6 and 12 hours, respectively. *P* value was calculated by unpaired Student’s t-test. (*p<0.05, **p<0.01, ***p<0.001).

**Supplementary Figure 2.** (**A**) L929, MDF, and HT29 cells were pretreated with DMSO, Vemurafenib (Vem), or Nec-1 followed by necroptosis induction for 12, 3, and 8 hours, respectively. The protein levels were determined by western blot. (**B**) HEK293T cells were transfected with RIPK3 for 48 hours. Then Vemurafenib or Nec-1 were added for 8 hours after transfection. The protein levels were determined by western blot.

**Supplementary Figure 3.** (**A**,**B**) MDF cells were transfected with siRNA targeting BRAF. After 48 hours, cells were pretreated with Vemurafenib (Vem) and Nec-1 followed by necroptosis induction. The protein levels (**A**) were determined by western blot. The cell viability (**B**) was determined by CellTiter-Glo® assay. *P* value was calculated by unpaired Student’s t-test. (*p<0.05, **p<0.01, ***p<0.001).

**Supplementary Figure 4.** (**A**,**B**) HEK293T cells were transfected with Flag-tagged wild-type (WT) or kinase domain of RIPK1 (residues 1–330) for 48 hours. After indicated treatment, the protein levels and phosphorylation of RIPK1 (**A**) were determined by western blot; the thermal stability of kinase domain of RIPK1 (**B**) was determined by CETSA assay. (**C**)The RMSD plot of Vemurafenib-RIPK1 complex during 100ns MD simulation trajectory. The red line represents Vemurafenib and the blue line represents RIPK1. (**D**) The RMSF plot of Vemurafenib-RIPK1 complex. (**E**) The potential allosteric binding pocket of RIPK1. The cyan cartoon structure represents RIPK1. The mesh structure represents the potential pocket and the stick structure represents the relative interaction residues. The last frames of 100ns MD simulations of Vemurafenib and Nec-1 binding position were shown by red and blue sticks. (**F**) The structural alignment of the initial (cyan cartoon) and final (yellow cartoon) conformations of Nec-1-bound RIPK1 during 100 ns MD simulations. The initial and last frames of 100ns MD simulations of Nec-1 binding position were shown by purple and blue sticks. (**G**) Alignment of the initial (cyan cartoon) and final (yellow cartoon) conformations of the helix (residues 37-66), beta-strand (residues 10-36), and activation loop (residues 156-196) of Nec-1-bound RIPK1. Movement of helix, beta-strand, and activation loop is indicated by arrow.

**Supplementary Table 1. Analysis of contributions of each residue in binding free energy.**
