# Supplementary figures and images for: Vemurafenib inhibits necroptosis in normal and pathological conditions as a RIPK1 antagonist
Source: Cell Death Dis. 2023 Aug 24;14(8):555. doi: 10.1038/s41419-023-06065-8 (PMC10449909; doi:10.1038/s41419-023-06065-8)

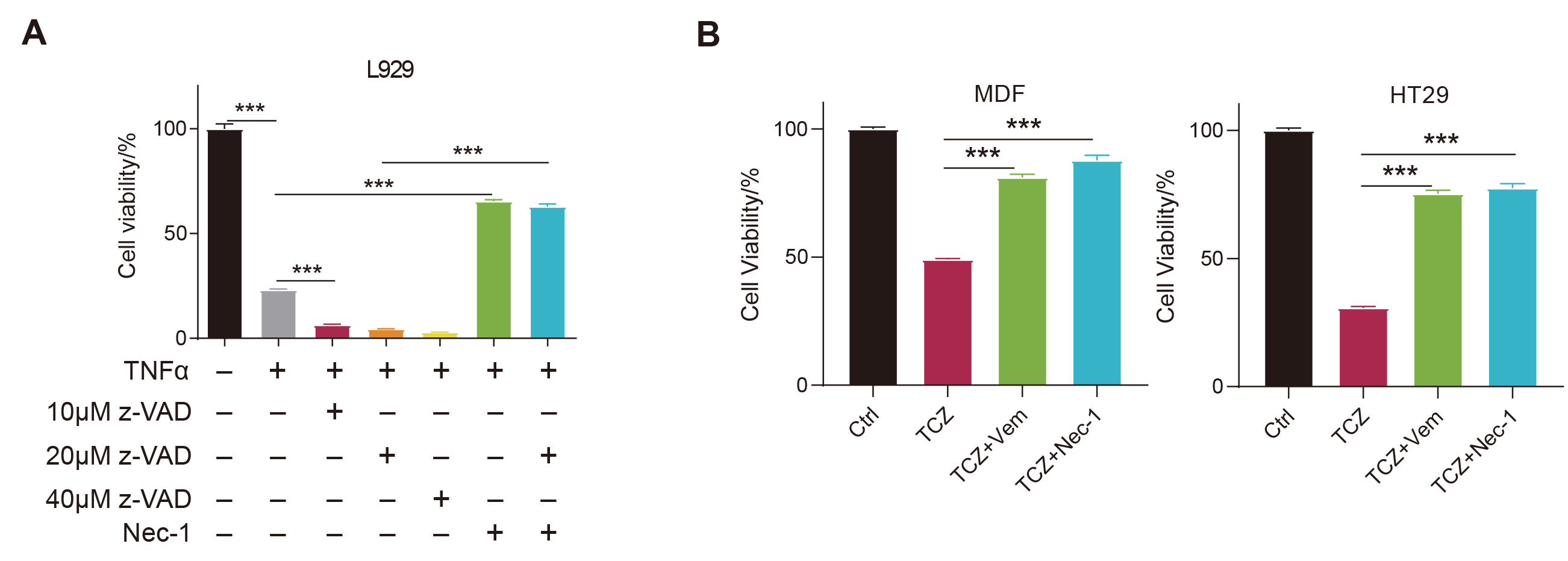

Supplement: Supplementary file 2 — Supplementary Figure 1 [file 41419_2023_6065_MOESM2_ESM.tif]

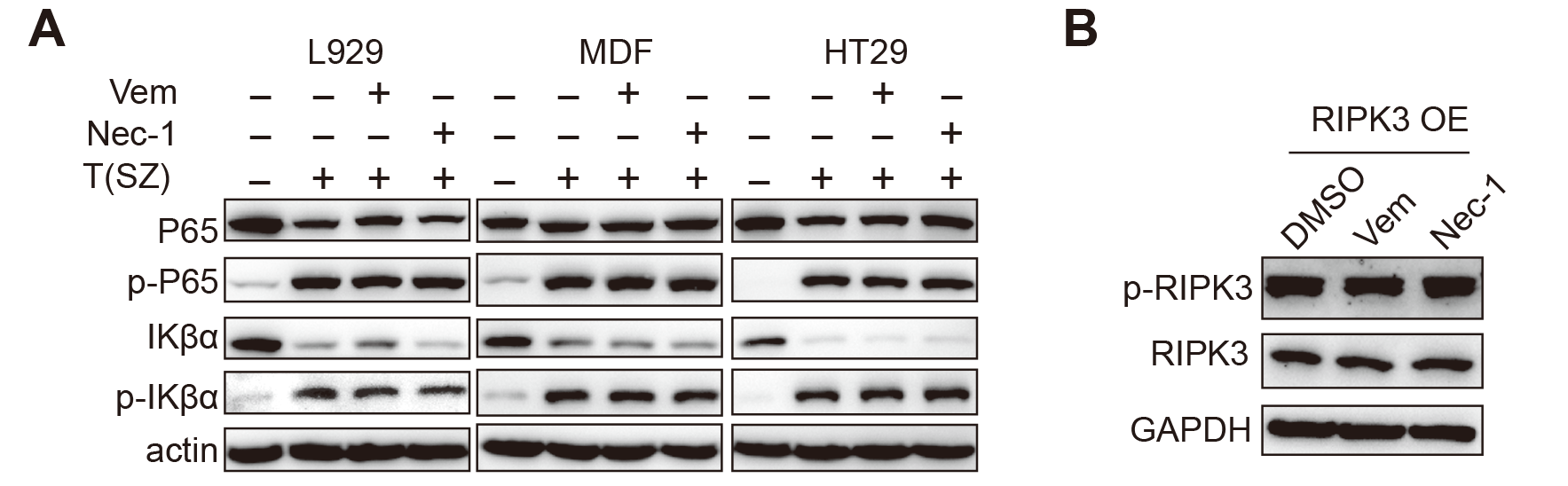

Supplement: Supplementary file 3 — Supplementary Figure 2 [file 41419_2023_6065_MOESM3_ESM.tif]

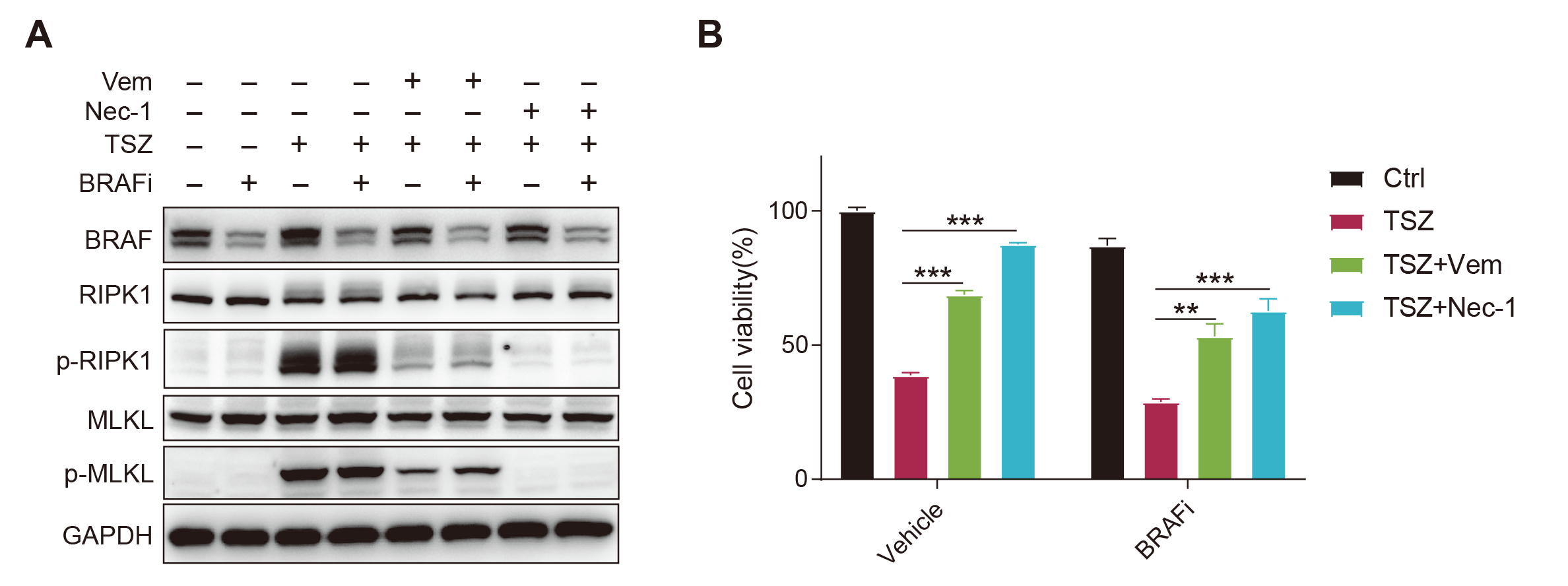

Supplement: Supplementary file 4 — Supplementary Figure 3 [file 41419_2023_6065_MOESM4_ESM.tif]

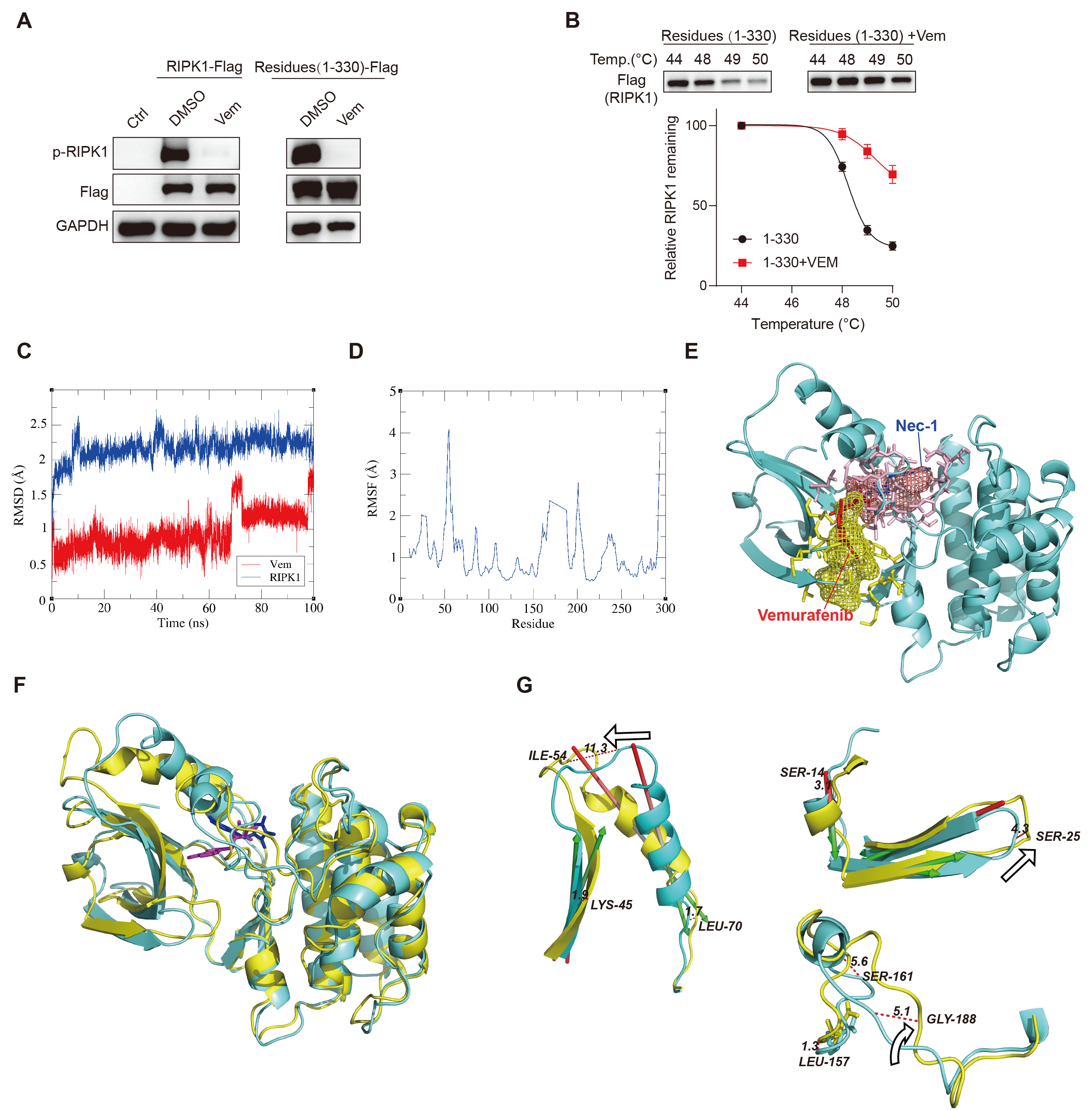

Supplement: Supplementary file 5 — Supplementary Figure 4 [file 41419_2023_6065_MOESM5_ESM.tif]
