# Supplementary material for: Vemurafenib inhibits necroptosis in normal and pathological conditions as a RIPK1 antagonist
Source: Cell Death Dis. 2023 Aug 24;14(8):555. doi: 10.1038/s41419-023-06065-8 (PMC10449909; doi:10.1038/s41419-023-06065-8)
Supplement: Supplementary file 6 — Supplementary table 1 [file 41419_2023_6065_MOESM6_ESM.docx]

**Supplementary Table 1. Analysis of contributions of each residue in binding free energy.**

| Residues | MMPBSA | MM | MM_COU | MM_VDW | PBSA | PBSA_PB | PBSA_SA |
| --- | --- | --- | --- | --- | --- | --- | --- |
| RIPK1-Vem | -134.142 | -262.080 | -38.002 | -224.078 | 127.938 | 154.529 | -26.590 |
| ILE-10 | 0.421 | -0.142 | -0.095 | -0.046 | 0.563 | 0.563 | 0.000 |
| LYS-11 | -0.046 | -0.545 | -0.532 | -0.013 | 0.500 | 0.500 | 0.000 |
| MET-12 | -0.078 | 0.029 | 0.059 | -0.030 | -0.107 | -0.107 | 0.000 |
| LYS-13 | -0.139 | -0.285 | -0.278 | -0.007 | 0.145 | 0.145 | 0.000 |
| SER-14 | -0.039 | -0.027 | -0.021 | -0.006 | -0.012 | -0.012 | 0.000 |
| SER-15 | -0.013 | -0.018 | -0.016 | -0.002 | 0.005 | 0.005 | 0.000 |
| ASP-16 | -0.136 | 0.121 | 0.126 | -0.005 | -0.257 | -0.256 | 0.000 |
| PHE-17 | 0.017 | 0.000 | 0.026 | -0.026 | 0.018 | 0.018 | 0.000 |
| LEU-18 | 0.022 | 0.015 | 0.019 | -0.004 | 0.007 | 0.007 | 0.000 |
| GLU-19 | -0.085 | -0.032 | -0.024 | -0.008 | -0.053 | -0.053 | 0.000 |
| SER-20 | 0.016 | -0.010 | 0.003 | -0.014 | 0.027 | 0.027 | 0.000 |
| ALA-21 | -0.065 | -0.010 | 0.018 | -0.028 | -0.055 | -0.055 | 0.000 |
| GLU-22 | -0.065 | 0.071 | 0.177 | -0.105 | -0.137 | -0.136 | 0.000 |
| LEU-23 | -1.798 | -1.865 | -0.038 | -1.827 | 0.067 | 0.170 | -0.103 |
| ASP-24 | -0.478 | -0.443 | -0.297 | -0.146 | -0.035 | -0.035 | 0.000 |
| SER-25 | 0.009 | -1.445 | -0.370 | -1.075 | 1.454 | 1.492 | -0.038 |
| GLY-29 | 1.511 | -2.912 | -0.292 | -2.620 | 4.422 | 4.767 | -0.345 |
| LYS-30 | -0.513 | -1.931 | 0.502 | -2.433 | 1.418 | 1.632 | -0.214 |
| VAL-31 | -7.046 | -7.308 | -0.070 | -7.238 | 0.261 | 0.997 | -0.736 |
| SER-32 | -0.799 | -0.666 | -0.224 | -0.441 | -0.133 | -0.133 | -0.001 |
| LEU-33 | -0.231 | -0.170 | 0.087 | -0.256 | -0.061 | -0.061 | 0.000 |
| ALA-34 | -0.106 | -0.092 | -0.053 | -0.039 | -0.014 | -0.014 | 0.000 |
| PHE-35 | -0.006 | 0.008 | 0.025 | -0.016 | -0.014 | -0.014 | 0.000 |
| HIS-36 | 0.011 | -0.056 | -0.043 | -0.014 | 0.068 | 0.068 | 0.000 |
| ARG-37 | 0.031 | -0.060 | -0.055 | -0.004 | 0.090 | 0.091 | 0.000 |
| THR-38 | -0.005 | -0.011 | -0.008 | -0.003 | 0.006 | 0.006 | 0.000 |
| GLN-39 | -0.067 | -0.072 | -0.059 | -0.013 | 0.005 | 0.005 | 0.000 |
| GLY-40 | -0.050 | -0.015 | -0.007 | -0.007 | -0.035 | -0.035 | 0.000 |
| LEU-41 | -0.043 | -0.103 | -0.052 | -0.051 | 0.060 | 0.060 | 0.000 |
| MET-42 | -0.084 | 0.078 | 0.228 | -0.150 | -0.162 | -0.162 | 0.000 |
| ILE-43 | -3.544 | -3.949 | 0.194 | -4.143 | 0.405 | 0.626 | -0.222 |
| MET-44 | -0.655 | -1.094 | -0.213 | -0.882 | 0.439 | 0.444 | -0.005 |
| LYS-45 | 3.518 | -12.152 | -4.597 | -7.554 | 15.669 | 16.703 | -1.033 |
| THR-46 | -0.880 | -0.406 | 0.075 | -0.481 | -0.473 | -0.470 | -0.003 |
| VAL-47 | -2.159 | -2.506 | -0.281 | -2.225 | 0.347 | 0.510 | -0.162 |
| TYR-48 | -0.210 | 0.010 | 0.114 | -0.104 | -0.219 | -0.219 | 0.000 |
| LYS-49 | -0.431 | -0.759 | -0.734 | -0.024 | 0.327 | 0.327 | 0.000 |
| GLY-50 | -0.033 | 0.013 | 0.016 | -0.004 | -0.046 | -0.046 | 0.000 |
| PRO-51 | -0.030 | 0.070 | 0.073 | -0.003 | -0.100 | -0.100 | 0.000 |
| ASN-52 | -0.023 | 0.028 | 0.031 | -0.003 | -0.052 | -0.052 | 0.000 |
| CYS-53 | -0.039 | 0.054 | 0.057 | -0.002 | -0.093 | -0.092 | 0.000 |
| ILE-54 | -0.029 | 0.034 | 0.036 | -0.003 | -0.063 | -0.063 | 0.000 |
| GLU-55 | 0.431 | 0.887 | 0.893 | -0.006 | -0.456 | -0.456 | 0.000 |
| HIS-56 | -0.102 | 0.083 | 0.101 | -0.018 | -0.186 | -0.185 | 0.000 |
| ASN-57 | -0.067 | 0.008 | 0.014 | -0.006 | -0.075 | -0.075 | 0.000 |
| GLU-58 | 0.135 | 0.540 | 0.548 | -0.008 | -0.405 | -0.405 | 0.000 |
| ALA-59 | -0.050 | 0.034 | 0.047 | -0.013 | -0.084 | -0.084 | 0.000 |
| LEU-60 | -0.263 | -0.235 | -0.068 | -0.167 | -0.028 | -0.027 | 0.000 |
| LEU-61 | -0.150 | -0.050 | -0.018 | -0.033 | -0.100 | -0.100 | 0.000 |
| GLU-62 | -0.154 | 0.322 | 0.341 | -0.019 | -0.476 | -0.476 | 0.000 |
| GLU-63 | -0.530 | 0.611 | 0.714 | -0.103 | -1.141 | -1.141 | 0.000 |
| ALA-64 | -0.363 | -0.443 | -0.272 | -0.171 | 0.080 | 0.080 | 0.000 |
| LYS-65 | 0.104 | -0.383 | -0.336 | -0.047 | 0.487 | 0.488 | 0.000 |
| MET-66 | -0.063 | -0.278 | -0.163 | -0.116 | 0.215 | 0.215 | 0.000 |
| MET-67 | -2.388 | -4.116 | -0.632 | -3.484 | 1.728 | 2.069 | -0.340 |
| ASN-68 | 0.136 | -0.188 | -0.010 | -0.178 | 0.323 | 0.323 | 0.000 |
| ARG-69 | 0.401 | 0.235 | 0.283 | -0.048 | 0.167 | 0.167 | 0.000 |
| LEU-70 | -0.346 | -0.417 | -0.017 | -0.400 | 0.071 | 0.112 | -0.040 |
| ARG-71 | 0.503 | 0.536 | 0.581 | -0.045 | -0.033 | -0.032 | 0.000 |
| HIS-72 | -0.040 | -0.198 | -0.161 | -0.037 | 0.159 | 0.159 | 0.000 |
| SER-73 | -0.007 | -0.053 | -0.036 | -0.016 | 0.046 | 0.046 | 0.000 |
| ARG-74 | 0.336 | 0.520 | 0.564 | -0.044 | -0.183 | -0.183 | 0.000 |
| VAL-75 | -0.700 | -0.355 | 0.195 | -0.549 | -0.345 | -0.288 | -0.057 |
| VAL-76 | -0.333 | -4.137 | -0.619 | -3.517 | 3.804 | 4.082 | -0.278 |
| LYS-77 | 0.680 | -0.326 | 0.995 | -1.320 | 1.006 | 1.015 | -0.009 |
| LEU-78 | -3.764 | -4.513 | -0.012 | -4.501 | 0.749 | 1.019 | -0.270 |
| LEU-79 | -0.059 | -0.209 | 0.053 | -0.262 | 0.150 | 0.150 | 0.000 |
| GLY-80 | -0.392 | -0.020 | 0.103 | -0.123 | -0.371 | -0.371 | 0.000 |
| VAL-81 | -0.070 | -0.312 | -0.187 | -0.125 | 0.242 | 0.242 | 0.000 |
| ILE-82 | -0.137 | 0.070 | 0.128 | -0.058 | -0.207 | -0.207 | 0.000 |
| ILE-83 | -0.012 | -0.041 | -0.024 | -0.016 | 0.028 | 0.029 | 0.000 |
| GLU-84 | 0.070 | 0.590 | 0.598 | -0.009 | -0.520 | -0.520 | 0.000 |
| GLU-85 | 0.289 | 0.486 | 0.490 | -0.004 | -0.197 | -0.197 | 0.000 |
| GLY-86 | -0.001 | 0.030 | 0.034 | -0.004 | -0.031 | -0.031 | 0.000 |
| LYS-87 | -0.136 | -0.918 | -0.893 | -0.025 | 0.782 | 0.783 | 0.000 |
| TYR-88 | -0.140 | 0.047 | 0.131 | -0.084 | -0.187 | -0.187 | 0.000 |
| SER-89 | -0.045 | -0.382 | -0.198 | -0.184 | 0.337 | 0.338 | 0.000 |
| LEU-90 | -3.754 | -5.429 | -0.676 | -4.753 | 1.675 | 2.019 | -0.344 |
| VAL-91 | -1.169 | -0.371 | 0.456 | -0.826 | -0.799 | -0.798 | -0.001 |
| MET-92 | -5.683 | -8.534 | -0.901 | -7.633 | 2.852 | 3.547 | -0.695 |
| GLU-93 | -1.037 | -2.483 | -1.921 | -0.562 | 1.446 | 1.447 | 0.000 |
| TYR-94 | -0.467 | -0.523 | -0.096 | -0.426 | 0.055 | 0.057 | -0.002 |
| MET-95 | 0.925 | -1.097 | 0.118 | -1.215 | 2.023 | 2.052 | -0.029 |
| GLU-96 | -0.105 | -0.279 | -0.111 | -0.167 | 0.173 | 0.174 | 0.000 |
| LYS-97 | -0.097 | -0.118 | 0.086 | -0.203 | 0.020 | 0.020 | 0.000 |
| GLY-98 | 1.155 | -1.411 | 0.152 | -1.562 | 2.565 | 2.643 | -0.078 |
| ASN-99 | -0.291 | -1.977 | 0.511 | -2.488 | 1.686 | 1.914 | -0.228 |
| LEU-100 | -0.189 | -0.098 | 0.018 | -0.116 | -0.091 | -0.090 | 0.000 |
| MET-101 | -0.028 | -0.002 | 0.103 | -0.105 | -0.026 | -0.026 | 0.000 |
| HIS-102 | -0.076 | -0.175 | 0.017 | -0.192 | 0.099 | 0.099 | 0.000 |
| VAL-103 | 0.003 | -0.020 | 0.023 | -0.043 | 0.023 | 0.023 | 0.000 |
| LEU-104 | 0.008 | 0.014 | 0.032 | -0.017 | -0.007 | -0.007 | 0.000 |
| LYS-105 | 0.444 | 0.518 | 0.543 | -0.025 | -0.074 | -0.074 | 0.000 |
| ALA-106 | -0.003 | -0.006 | 0.000 | -0.006 | 0.003 | 0.003 | 0.000 |
| GLU-107 | -0.194 | -0.195 | -0.193 | -0.003 | 0.002 | 0.002 | 0.000 |
| MET-108 | -0.011 | -0.011 | -0.008 | -0.004 | 0.000 | 0.001 | 0.000 |
| SER-109 | 0.000 | 0.003 | 0.004 | -0.002 | -0.002 | -0.002 | 0.000 |
| THR-110 | 0.001 | -0.001 | 0.002 | -0.003 | 0.002 | 0.003 | 0.000 |
| PRO-111 | -0.007 | -0.014 | -0.013 | -0.001 | 0.007 | 0.007 | 0.000 |
| LEU-112 | -0.005 | -0.011 | -0.010 | -0.001 | 0.006 | 0.006 | 0.000 |
| SER-113 | -0.006 | -0.011 | -0.010 | -0.001 | 0.005 | 0.006 | 0.000 |
| VAL-114 | -0.006 | -0.015 | -0.012 | -0.003 | 0.009 | 0.009 | 0.000 |
| LYS-115 | 0.201 | 0.250 | 0.253 | -0.003 | -0.049 | -0.049 | 0.000 |
| GLY-116 | -0.004 | -0.013 | -0.012 | -0.001 | 0.009 | 0.009 | 0.000 |
| ARG-117 | 0.252 | 0.302 | 0.307 | -0.005 | -0.050 | -0.050 | 0.000 |
| ILE-118 | -0.013 | -0.033 | -0.023 | -0.010 | 0.021 | 0.021 | 0.000 |
| ILE-119 | -0.008 | -0.027 | -0.023 | -0.004 | 0.019 | 0.019 | 0.000 |
| LEU-120 | -0.008 | -0.019 | -0.016 | -0.003 | 0.011 | 0.011 | 0.000 |
| GLU-121 | -0.325 | -0.526 | -0.515 | -0.011 | 0.201 | 0.202 | 0.000 |
| ILE-122 | -0.031 | -0.049 | -0.032 | -0.017 | 0.018 | 0.018 | 0.000 |
| ILE-123 | -0.012 | -0.027 | -0.022 | -0.005 | 0.015 | 0.015 | 0.000 |
| GLU-124 | -0.372 | -0.497 | -0.491 | -0.006 | 0.126 | 0.126 | 0.000 |
| GLY-125 | -0.038 | -0.028 | -0.017 | -0.012 | -0.010 | -0.009 | 0.000 |
| MET-126 | -0.049 | -0.084 | -0.059 | -0.025 | 0.035 | 0.035 | 0.000 |
| ALA-127 | -0.003 | 0.008 | 0.012 | -0.004 | -0.010 | -0.010 | 0.000 |
| TYR-128 | -0.029 | -0.017 | 0.018 | -0.035 | -0.012 | -0.012 | 0.000 |
| LEU-129 | -0.090 | -0.041 | 0.064 | -0.105 | -0.050 | -0.042 | -0.008 |
| HIS-130 | -0.016 | 0.032 | 0.041 | -0.009 | -0.048 | -0.048 | 0.000 |
| GLY-131 | 0.016 | 0.033 | 0.036 | -0.002 | -0.017 | -0.017 | 0.000 |
| LYS-132 | 0.452 | 0.580 | 0.596 | -0.016 | -0.128 | -0.128 | 0.000 |
| GLY-133 | 0.016 | 0.026 | 0.030 | -0.004 | -0.010 | -0.010 | 0.000 |
| VAL-134 | -0.044 | -0.044 | -0.001 | -0.043 | 0.000 | 0.001 | -0.001 |
| ILE-135 | -0.033 | -0.034 | -0.012 | -0.023 | 0.002 | 0.002 | 0.000 |
| HIS-136 | -0.090 | 0.043 | 0.231 | -0.189 | -0.132 | -0.132 | 0.000 |
| LYS-137 | -0.228 | -0.129 | -0.093 | -0.036 | -0.099 | -0.098 | 0.000 |
| ASP-138 | 0.655 | -0.387 | -0.241 | -0.146 | 1.042 | 1.042 | 0.000 |
| LEU-139 | -0.056 | -0.012 | 0.039 | -0.052 | -0.044 | -0.044 | 0.000 |
| LYS-140 | -0.432 | 0.129 | 0.339 | -0.210 | -0.561 | -0.552 | -0.009 |
| PRO-141 | -0.139 | -0.127 | -0.032 | -0.094 | -0.012 | -0.012 | 0.000 |
| GLU-142 | 0.629 | -2.295 | -0.673 | -1.622 | 2.924 | 3.032 | -0.108 |
| ASN-143 | -0.696 | -0.792 | -0.035 | -0.757 | 0.096 | 0.097 | -0.001 |
| ILE-144 | -0.409 | -0.322 | -0.015 | -0.307 | -0.087 | -0.087 | 0.000 |
| LEU-145 | -4.407 | -4.375 | -0.161 | -4.214 | -0.032 | 0.262 | -0.294 |
| VAL-146 | 0.020 | -0.155 | 0.071 | -0.226 | 0.175 | 0.175 | 0.000 |
| ASP-147 | -0.263 | -0.362 | -0.310 | -0.052 | 0.099 | 0.099 | 0.000 |
| ASN-148 | -0.017 | -0.011 | 0.004 | -0.015 | -0.006 | -0.006 | 0.000 |
| ASP-149 | -0.257 | -0.271 | -0.265 | -0.006 | 0.014 | 0.014 | 0.000 |
| PHE-150 | -0.021 | -0.027 | -0.012 | -0.014 | 0.005 | 0.006 | 0.000 |
| HIS-151 | 0.022 | 0.022 | 0.040 | -0.018 | 0.000 | 0.000 | 0.000 |
| ILE-152 | -0.088 | -0.057 | -0.009 | -0.048 | -0.031 | -0.030 | 0.000 |
| LYS-153 | 0.287 | 1.028 | 1.226 | -0.198 | -0.741 | -0.741 | 0.000 |
| ILE-154 | -0.297 | -0.690 | -0.204 | -0.486 | 0.392 | 0.396 | -0.004 |
| ALA-155 | -1.590 | -2.387 | 0.188 | -2.574 | 0.797 | 0.854 | -0.057 |
| ASP-156 | 6.064 | -13.263 | -9.310 | -3.953 | 19.326 | 19.803 | -0.477 |
| LEU-157 | -9.700 | -15.401 | 0.110 | -15.511 | 5.701 | 7.227 | -1.526 |
| GLY-158 | -1.958 | -2.175 | -0.048 | -2.127 | 0.217 | 0.280 | -0.062 |
| LEU-159 | -7.433 | -7.562 | -0.471 | -7.091 | 0.129 | 0.652 | -0.523 |
| ALA-160 | -0.426 | -0.272 | -0.009 | -0.263 | -0.154 | -0.154 | 0.000 |
| SER-161 | -0.060 | -1.159 | -0.749 | -0.409 | 1.099 | 1.114 | -0.015 |
| PHE-162 | -1.268 | -1.879 | -0.250 | -1.629 | 0.611 | 0.681 | -0.070 |
| LYS-163 | -0.222 | -0.941 | -0.913 | -0.029 | 0.719 | 0.719 | 0.000 |
| MET-164 | -0.038 | -0.190 | -0.127 | -0.062 | 0.152 | 0.152 | 0.000 |
| TRP-165 | -0.480 | -0.338 | 0.370 | -0.708 | -0.142 | -0.116 | -0.026 |
| SER-166 | -0.005 | -0.103 | -0.073 | -0.030 | 0.098 | 0.098 | 0.000 |
| LYS-167 | -0.476 | -0.854 | -0.837 | -0.017 | 0.378 | 0.379 | 0.000 |
| LEU-168 | -0.103 | -0.145 | -0.047 | -0.098 | 0.042 | 0.046 | -0.004 |
| ASN-169 | -0.016 | 0.004 | 0.040 | -0.036 | -0.020 | -0.018 | -0.002 |
| GLY-188 | -0.042 | 0.002 | 0.018 | -0.016 | -0.044 | -0.044 | 0.000 |
| THR-189 | -0.050 | -0.021 | 0.020 | -0.041 | -0.029 | -0.029 | 0.000 |
| LEU-190 | -0.010 | -0.014 | -0.005 | -0.009 | 0.004 | 0.005 | 0.000 |
| TYR-191 | -0.028 | -0.020 | -0.005 | -0.015 | -0.009 | -0.008 | 0.000 |
| TYR-192 | -0.030 | -0.033 | -0.008 | -0.025 | 0.004 | 0.004 | 0.000 |
| MET-193 | -0.023 | -0.013 | -0.006 | -0.007 | -0.010 | -0.010 | 0.000 |
| ALA-194 | 0.008 | 0.010 | 0.012 | -0.002 | -0.003 | -0.003 | 0.000 |
| PRO-195 | 0.006 | 0.008 | 0.009 | -0.001 | -0.001 | -0.001 | 0.000 |
| GLU-196 | -0.059 | -0.116 | -0.114 | -0.001 | 0.057 | 0.057 | 0.000 |
| HIS-197 | 0.007 | 0.010 | 0.013 | -0.003 | -0.003 | -0.003 | 0.000 |
| LEU-198 | -0.004 | 0.003 | 0.005 | -0.002 | -0.006 | -0.006 | 0.000 |
| ASN-199 | -0.001 | 0.002 | 0.003 | -0.001 | -0.004 | -0.004 | 0.000 |
| ASP-200 | 0.041 | 0.027 | 0.028 | -0.001 | 0.014 | 0.014 | 0.000 |
| VAL-201 | 0.008 | 0.007 | 0.008 | -0.001 | 0.000 | 0.000 | 0.000 |
| ASN-202 | 0.007 | 0.007 | 0.008 | -0.001 | 0.000 | 0.000 | 0.000 |
| ALA-203 | 0.012 | 0.011 | 0.012 | -0.001 | 0.001 | 0.001 | 0.000 |
| LYS-204 | -0.125 | -0.108 | -0.106 | -0.002 | -0.017 | -0.017 | 0.000 |
| PRO-205 | -0.002 | 0.011 | 0.015 | -0.004 | -0.013 | -0.013 | 0.000 |
| THR-206 | 0.004 | -0.018 | -0.016 | -0.002 | 0.022 | 0.022 | 0.000 |
| GLU-207 | -0.159 | -0.217 | -0.215 | -0.003 | 0.059 | 0.059 | 0.000 |
| LYS-208 | 0.119 | 0.156 | 0.158 | -0.002 | -0.037 | -0.037 | 0.000 |
| SER-209 | -0.002 | -0.002 | 0.000 | -0.003 | 0.000 | 0.000 | 0.000 |
| ASP-210 | -0.085 | -0.446 | -0.435 | -0.010 | 0.361 | 0.361 | 0.000 |
| VAL-211 | -0.004 | -0.012 | -0.009 | -0.003 | 0.008 | 0.008 | 0.000 |
| TYR-212 | -0.012 | -0.005 | -0.001 | -0.004 | -0.007 | -0.007 | 0.000 |
| SER-213 | -0.011 | -0.003 | 0.004 | -0.007 | -0.008 | -0.008 | 0.000 |
| PHE-214 | -0.010 | -0.008 | 0.000 | -0.007 | -0.002 | -0.002 | 0.000 |
| ALA-215 | -0.007 | -0.004 | -0.002 | -0.002 | -0.003 | -0.003 | 0.000 |
| VAL-216 | -0.015 | -0.002 | 0.003 | -0.005 | -0.013 | -0.013 | 0.000 |
| VAL-217 | -0.023 | -0.004 | 0.008 | -0.012 | -0.019 | -0.019 | 0.000 |
| LEU-218 | -0.008 | -0.003 | 0.000 | -0.003 | -0.005 | -0.005 | 0.000 |
| TRP-219 | -0.010 | -0.006 | -0.003 | -0.004 | -0.004 | -0.004 | 0.000 |
| ALA-220 | -0.010 | 0.003 | 0.007 | -0.004 | -0.013 | -0.012 | 0.000 |
| ILE-221 | -0.009 | -0.001 | 0.005 | -0.006 | -0.008 | -0.008 | 0.000 |
| PHE-222 | -0.003 | 0.002 | 0.004 | -0.002 | -0.005 | -0.005 | 0.000 |
| ALA-223 | -0.002 | 0.006 | 0.007 | -0.001 | -0.007 | -0.007 | 0.000 |
| ASN-224 | -0.011 | -0.016 | -0.012 | -0.004 | 0.005 | 0.005 | 0.000 |
| LYS-225 | 0.190 | 0.219 | 0.224 | -0.005 | -0.029 | -0.029 | 0.000 |
| GLU-226 | -0.229 | -0.451 | -0.432 | -0.018 | 0.222 | 0.222 | 0.000 |
| PRO-227 | -0.006 | -0.009 | -0.006 | -0.003 | 0.004 | 0.004 | 0.000 |
| TYR-228 | -0.014 | -0.016 | -0.013 | -0.002 | 0.001 | 0.001 | 0.000 |
| GLU-234 | -0.133 | -0.148 | -0.146 | -0.003 | 0.015 | 0.015 | 0.000 |
| GLN-235 | -0.003 | -0.007 | 0.005 | -0.012 | 0.003 | 0.004 | 0.000 |
| GLN-236 | 0.006 | 0.006 | 0.008 | -0.003 | 0.000 | 0.000 | 0.000 |
| LEU-237 | 0.001 | 0.003 | 0.004 | -0.001 | -0.002 | -0.001 | 0.000 |
| ILE-238 | -0.002 | -0.002 | 0.001 | -0.003 | -0.001 | 0.000 | 0.000 |
| MET-239 | 0.000 | -0.001 | 0.000 | -0.001 | 0.001 | 0.001 | 0.000 |
| ALA-240 | 0.001 | 0.002 | 0.003 | 0.000 | -0.002 | -0.001 | 0.000 |
| ILE-241 | -0.003 | 0.000 | 0.002 | -0.001 | -0.003 | -0.003 | 0.000 |
| LYS-242 | 0.015 | 0.036 | 0.037 | -0.001 | -0.021 | -0.021 | 0.000 |
| SER-243 | 0.001 | 0.002 | 0.002 | 0.000 | -0.001 | -0.001 | 0.000 |
| GLY-244 | -0.002 | -0.001 | -0.001 | 0.000 | -0.001 | -0.001 | 0.000 |
| ASN-245 | 0.000 | -0.006 | -0.005 | -0.001 | 0.005 | 0.005 | 0.000 |
| ARG-246 | 0.095 | 0.141 | 0.142 | -0.001 | -0.047 | -0.046 | 0.000 |
| PRO-247 | 0.001 | 0.007 | 0.008 | -0.001 | -0.006 | -0.006 | 0.000 |
| ASP-248 | -0.132 | -0.150 | -0.150 | 0.000 | 0.018 | 0.018 | 0.000 |
| VAL-249 | -0.002 | -0.002 | -0.002 | 0.000 | 0.000 | 0.000 | 0.000 |
| ASP-250 | -0.127 | -0.131 | -0.131 | 0.000 | 0.005 | 0.005 | 0.000 |
| ASP-251 | -0.157 | -0.176 | -0.176 | -0.001 | 0.019 | 0.019 | 0.000 |
| ILE-252 | 0.003 | 0.008 | 0.009 | -0.001 | -0.005 | -0.005 | 0.000 |
| THR-253 | 0.002 | 0.004 | 0.005 | -0.001 | -0.003 | -0.003 | 0.000 |
| GLU-254 | -0.192 | -0.226 | -0.225 | -0.001 | 0.034 | 0.034 | 0.000 |
| TYR-255 | -0.004 | -0.006 | -0.006 | -0.001 | 0.003 | 0.003 | 0.000 |
| CYS-256 | 0.002 | 0.004 | 0.005 | 0.000 | -0.002 | -0.002 | 0.000 |
| PRO-257 | -0.002 | -0.005 | -0.005 | 0.000 | 0.003 | 0.003 | 0.000 |
| ARG-258 | 0.126 | 0.132 | 0.132 | 0.000 | -0.006 | -0.005 | 0.000 |
| GLU-259 | -0.147 | -0.161 | -0.161 | 0.000 | 0.014 | 0.014 | 0.000 |
| ILE-260 | 0.000 | -0.003 | -0.002 | -0.001 | 0.003 | 0.003 | 0.000 |
| ILE-261 | -0.001 | -0.004 | -0.003 | 0.000 | 0.003 | 0.003 | 0.000 |
| SER-262 | 0.002 | 0.001 | 0.001 | 0.000 | 0.001 | 0.001 | 0.000 |
| LEU-263 | 0.002 | 0.001 | 0.002 | -0.001 | 0.001 | 0.001 | 0.000 |
| MET-264 | 0.002 | 0.000 | 0.001 | -0.001 | 0.002 | 0.002 | 0.000 |
| LYS-265 | 0.125 | 0.140 | 0.140 | 0.000 | -0.015 | -0.015 | 0.000 |
| LEU-266 | 0.003 | 0.004 | 0.004 | -0.001 | -0.001 | -0.001 | 0.000 |
| CYS-267 | 0.001 | -0.002 | 0.000 | -0.001 | 0.002 | 0.003 | 0.000 |
| TRP-268 | 0.000 | -0.002 | -0.001 | -0.002 | 0.002 | 0.002 | 0.000 |
| GLU-269 | -0.104 | -0.129 | -0.128 | -0.001 | 0.025 | 0.025 | 0.000 |
| ALA-270 | 0.001 | -0.001 | 0.000 | 0.000 | 0.002 | 0.002 | 0.000 |
| ASN-271 | -0.003 | -0.007 | -0.007 | 0.000 | 0.004 | 0.004 | 0.000 |
| PRO-272 | -0.006 | -0.008 | -0.008 | -0.001 | 0.002 | 0.002 | 0.000 |
| GLU-273 | -0.087 | -0.094 | -0.093 | 0.000 | 0.007 | 0.007 | 0.000 |
| ALA-274 | -0.004 | -0.003 | -0.003 | 0.000 | -0.001 | -0.001 | 0.000 |
| ARG-275 | 0.088 | 0.166 | 0.167 | -0.001 | -0.077 | -0.077 | 0.000 |
| PRO-276 | 0.005 | -0.002 | -0.001 | -0.001 | 0.006 | 0.006 | 0.000 |
| THR-277 | 0.001 | 0.007 | 0.008 | -0.001 | -0.006 | -0.006 | 0.000 |
| PHE-278 | -0.005 | 0.001 | 0.007 | -0.006 | -0.007 | -0.006 | 0.000 |
| PRO-279 | 0.002 | 0.002 | 0.003 | -0.001 | 0.000 | 0.000 | 0.000 |
| GLY-280 | 0.000 | 0.005 | 0.005 | 0.000 | -0.005 | -0.005 | 0.000 |
| ILE-281 | 0.000 | 0.009 | 0.010 | -0.001 | -0.009 | -0.009 | 0.000 |
| GLU-282 | -0.279 | -0.337 | -0.335 | -0.001 | 0.058 | 0.058 | 0.000 |
| GLU-283 | -0.186 | -0.195 | -0.194 | 0.000 | 0.009 | 0.009 | 0.000 |
| LYS-284 | 0.162 | 0.181 | 0.181 | 0.000 | -0.019 | -0.019 | 0.000 |
| PHE-285 | 0.002 | 0.005 | 0.006 | -0.001 | -0.003 | -0.003 | 0.000 |
| ARG-286 | 0.281 | 0.334 | 0.336 | -0.001 | -0.054 | -0.053 | 0.000 |
| PRO-287 | 0.000 | 0.001 | 0.001 | 0.000 | -0.001 | 0.000 | 0.000 |
| PHE-288 | 0.002 | 0.005 | 0.006 | -0.001 | -0.003 | -0.003 | 0.000 |
| TYR-289 | 0.001 | 0.003 | 0.004 | -0.001 | -0.002 | -0.001 | 0.000 |
| LEU-290 | 0.002 | 0.003 | 0.003 | 0.000 | -0.001 | -0.001 | 0.000 |
| SER-291 | 0.003 | 0.004 | 0.005 | 0.000 | -0.002 | -0.001 | 0.000 |
| GLN-292 | 0.001 | 0.001 | 0.002 | 0.000 | 0.000 | 0.000 | 0.000 |
| LEU-293 | 0.001 | 0.002 | 0.002 | -0.001 | -0.001 | -0.001 | 0.000 |
| GLU-294 | -0.332 | -0.352 | -0.351 | 0.000 | 0.020 | 0.020 | 0.000 |
