# Supplementary material for: Vemurafenib inhibits necroptosis in normal and pathological conditions as a RIPK1 antagonist
Source: Cell Death Dis. 2023 Aug 24;14(8):555. doi: 10.1038/s41419-023-06065-8 (PMC10449909; doi:10.1038/s41419-023-06065-8)

Fig 2A

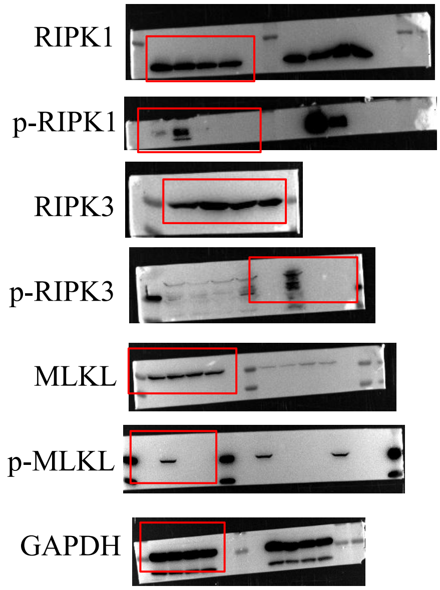

Fig 2B

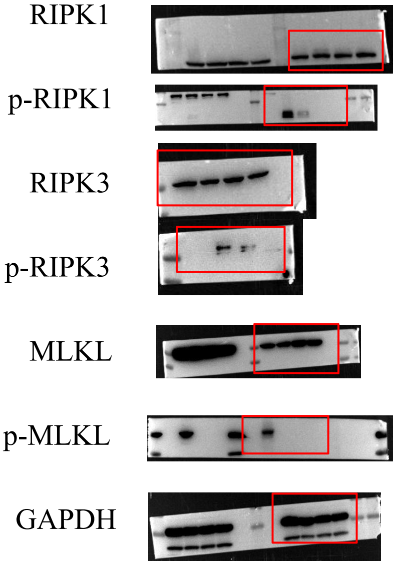

Fig 2C

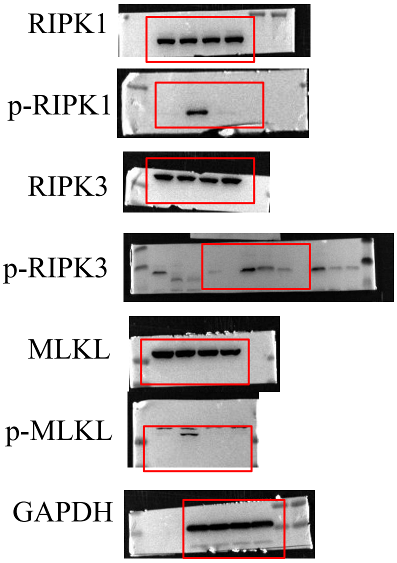

Fig 2D

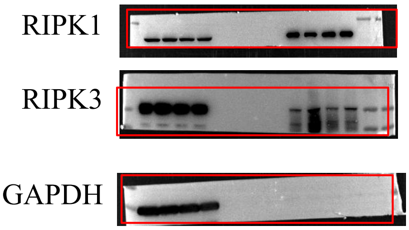

Fig 2E

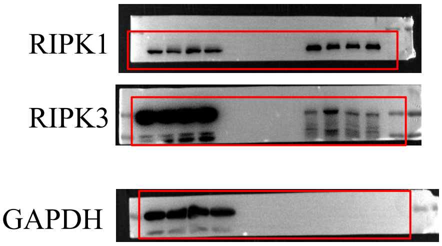

Fig 2F

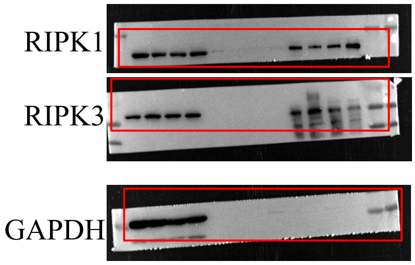

Fig 2G

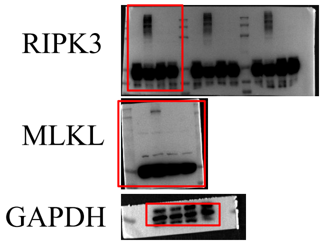

Fig 2H

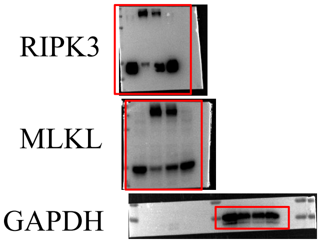

Fig 2I

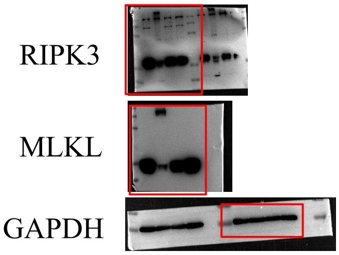

Fig 2J

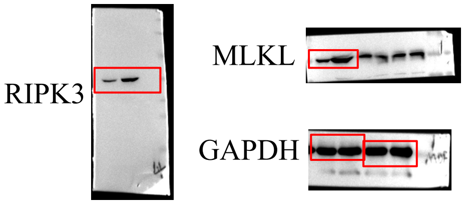

Fig 3C

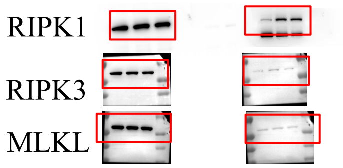

Fig 3D

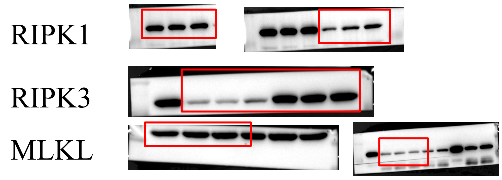

Fig 3E

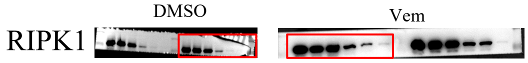

Fig 3F

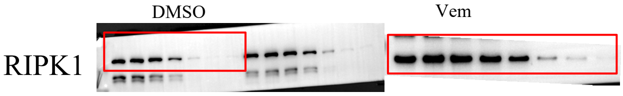

Fig 4E

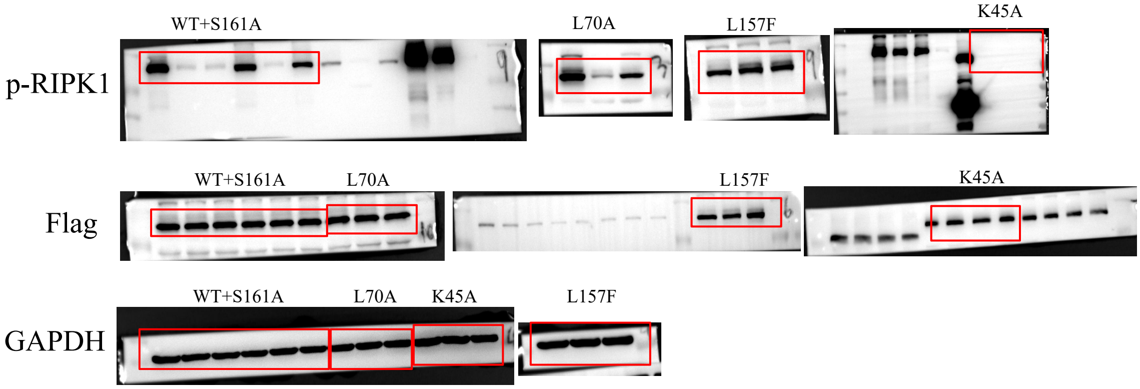

Fig 4F

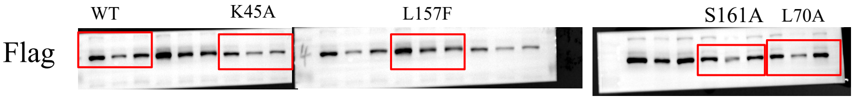

Fig 4G

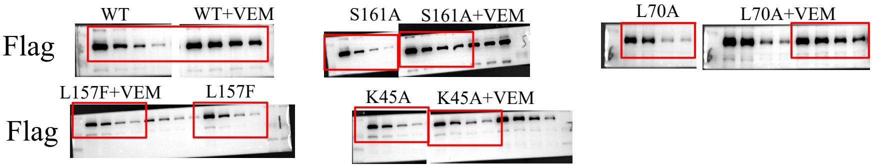

Supplementary figure

Fig S2A

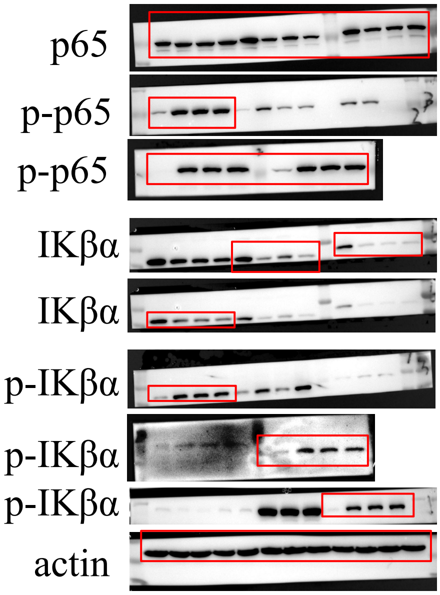

Fig S2B

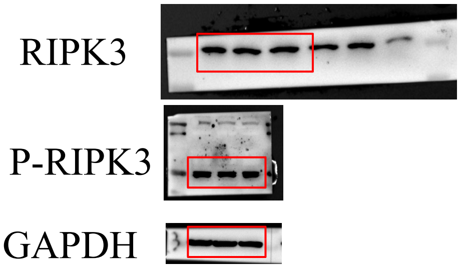

Fig S3A

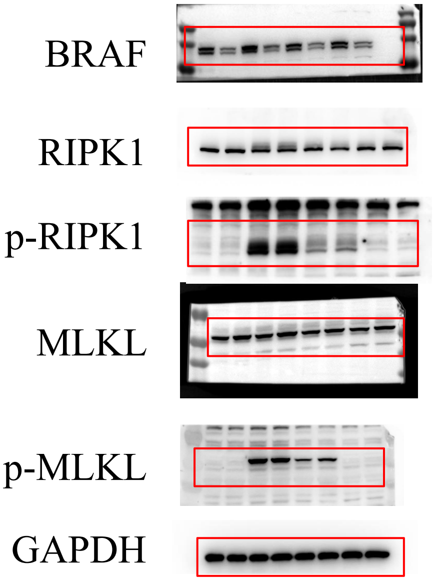

Fig S4A

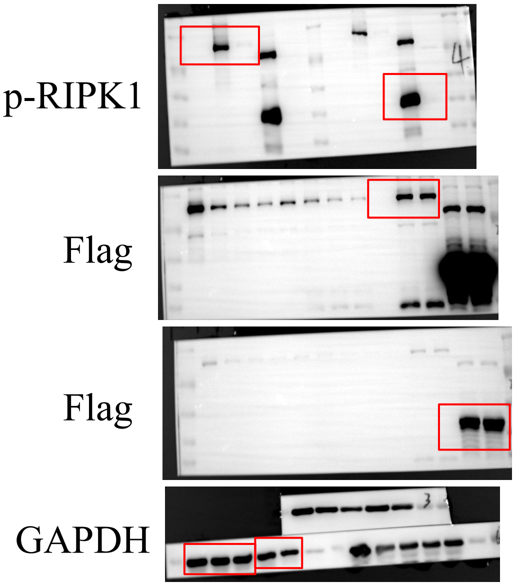

Fig S4B

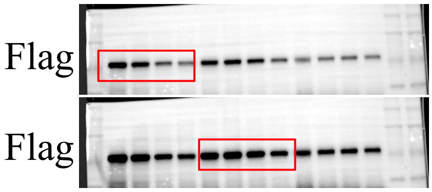

Supplement: Supplementary file 7 — original western blots [file 41419_2023_6065_MOESM7_ESM.pdf]
